# Marginal zone B cells exacerbate endotoxic shock via interleukin-6 secretion induced by Fcα/μR-coupled TLR4 signalling

**DOI:** 10.1038/ncomms11498

**Published:** 2016-05-05

**Authors:** Shin-ichiro Honda, Kazuki Sato, Naoya Totsuka, Satoshi Fujiyama, Manabu Fujimoto, Kensuke Miyake, Chigusa Nakahashi-Oda, Satoko Tahara-Hanaoka, Kazuko Shibuya, Akira Shibuya

**Affiliations:** 1 Department of Immunology, Faculty of Medicine, University of Tsukuba, 1-1-1, Tennohdai, Tsukuba 305-8575, Ibaraki, Japan; 2 Department of Dermatology, Faculty of Medicine, University of Tsukuba, 1-1-1, Tennohdai, Tsukuba 305-8575, Ibaraki, Japan; 3 Division of Innate Immunity, Institute of Medical Sciences, University of Tokyo, Shirokanedai, Minatoloku, Tokyo 108-8639, Japan; 4 Life Science Center of Tsukuba Advanced Research Alliance (TARA), University of Tsukuba, 1-1-1, Tennohdai, Tsukuba 305-8575, Ibaraki, Japan

## Abstract

Marginal zone (MZ) B cells produce a first wave of antibodies for protection from blood-borne pathogens. However, the role of MZ B cells in inflammatory responses has not been elucidated. Here we show that MZ B cells produce pro-inflammatory cytokines, such as interleukin-6 (IL-6), and exacerbate systemic inflammatory responses to lipopolysaccharide (LPS). After intravenous injection of LPS or *E. coli*, mice deficient in MZ B cells or IL-6 only in MZ B cells have attenuated systemic inflammatory responses and prolonged survival compared with wild-type mice. LPS directly stimulates MZ B cells via Toll-like receptor 4 (TLR4) and MyD88 pathways for IL-6 production. Furthermore, TLR4 requires physical and functional association with Fcα/μR (CD351) for its oligomer formation, NF-κB signalling and IL-6 production from MZ B cells; this association is responsible for systemic inflammatory responses and endotoxic shock. These results reveal a pro-inflammatory role of MZ B cells in endotoxic shock.

## Abstract (web summary)

Marginal zone B cells are mostly characterized in the context of host defense against bacterial blood-borne pathogens. Here the authors show that TLR4 signaling in these cells requires Fcα/μR (CD351) and that they are a major source of IL-6 in a mouse model of sepsis.

## Graphical abstract

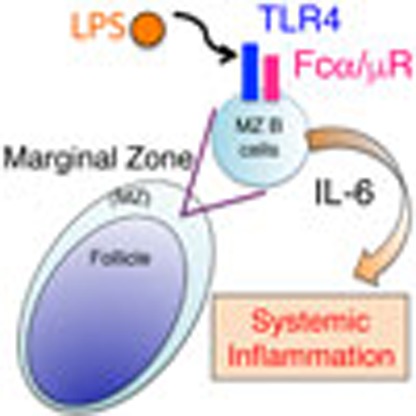

Sepsis is one major cause of systemic inflammatory response syndrome (SIRS), which sometimes leads to host death^1^. Many factors such as bacterial products (pathogen-associated molecular patterns) and those released from damaged cells (damage-associated molecular patterns) are known to trigger SIRS^2^. During SIRS caused by lipopolysaccharides (LPS) of Gram-negative bacteria, Toll-like receptor 4 (TLR4), which initiates the production of inflammatory cytokines and chemokines, has been thought to be pivotal in pathophysiology of sepsis^3^.

Marginal zone (MZ) segregates the circulating blood from the lymphoid tissues in the spleen and contains several types of immune cells including MZ B cells. MZ B cells express B-cell antigen receptors poly-reactive to various pathogens with low affinity^4^. After encountering blood-borne pathogens, MZ B cells collaborate with dendritic cells^5^ and neutrophils^6^ to rapidly produce a first wave innate-like antibodies^7^,^8^, which plays an important role in eradication of pathogens^9^,^10^. Indeed, mice deficient in MZ B cells showed decreased antibody production in the early phase after pathogen invasion into the blood circulation^11^,^12^. However, the involvement of MZ B cells in inflammatory responses has not been elucidated.

Fc receptors (FcRs) play critical roles in immune responses, including inflammation, cytotoxicity and allergic reactions^13^,^14^. Fcα/μR (CD351) is an FcR for IgA and IgM^15^,^16^. *Feamr* gene is located near the clusters for IgG FcRs on chromosome 1 (refs 15, 17, 18). The cytoplasmic region of Fcα/μR is required for an atypical dimer formation^19^,^20^. Fcα/μR is preferentially expressed on follicular dendritic cells in the lymphoid organs^21^ and suppresses *T*-independent antigen retention by follicular dendritic cells, leading to the downregulation of germinal centre formation and humoral immune responses, including antibody production, affinity maturation and memory B-cell generation, against *T*-independent antigens^21^. Fcα/μR is also expressed on MZ B cells. However, the functional role of Fcα/μR on MZ B cells has remained unclear.

Here we investigate the role of MZ B cells in systemic inflammatory responses during endotoxic shock. We report that MZ B cells produce interleukin-6 (IL-6) in response to LPS via the TLR4 and NF-κB signalling pathways and exacerbate endotoxic shock. We also demonstrate that Fcα/μR physically and functionally associates with TLR4 and induces the oligomer formation of TLR4 for amplification of IL-6 production.

## Results

### Mice lacking MZ B cells are resistant to endotoxic shock

**Figure 1:**
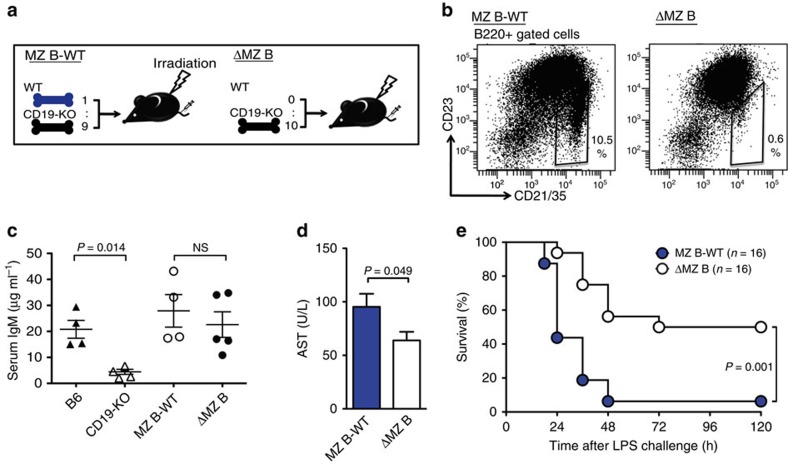
MZ B cells contribute to inflammatory response against endotoxic shock. (**a**) Strategy to generate mice lacking MZ B cells (ΔMZ B) or control mice (MZ B-WT). Lethally irradiated mice received i.v. injection of mixed bone marrow cells from WT and CD19-deficient mice at a ratio indicated. (**b**) Flow cytometry of splenocytes. Percentage of MZ B cells among B cell population were shown. (**c**) Natural IgM in sera of mice indicated were measured by enzyme-linked immunosorbant assay. (**d**,**e**) ΔMZ B or MZ B-WT mice were i.v. injected with LPS. Mice were then analysed for serum AST level 12 h after CLP (**d**) and monitored for survival rate of mice (**e**). Data are representative of three independent experiments. For survival analysis, data are pooled of each experiment and the total numbers of mice are indicated. Statistical analyses were performed with the unpaired Student's *t*-test. The log-rank test was used for mice survival. Error bars indicate s.d. NS, not significant.

To examine the role of MZ B cells in inflammatory responses, we generated MZ B-cell-deficient bone marrow (BM) chimeric mice (ΔMZ B) by transferring *Cd19*^−/−^ BM cells into lethally irradiated mice (Fig. 1a). As a control, we also generated BM chimeric mice (MZ B-WT) by transferring wild-type (WT) and *Cd19*^−/−^ BM cells at a ratio of 1:9, respectively, after lethal irradiation (Fig. 1a). ΔMZ B mice showed significantly lower number of MZ B cells, compared with MZ B-WT mice (Fig. 1b; Supplementary Table 1). However, the development of immune cells, including immature and mature B-cell subsets other than MZ B cells, was comparable between ΔMZ B and MZ B-WT mice (Supplementary Table 1). *Cd19*^−/−^ mice lack natural IgM, a critical component for LPS clearance^22^, as a result of defective development of peritoneal B1 B cells^23^. Despite this, both ΔMZ B and MZ B-WT mice had comparable amounts of serum natural IgM (Fig. 1c). After intravenous (i.v.) injection of LPS (600 μg per mouse), ΔMZ B mice had attenuated liver dysfunction compared with MZ B-WT mice (Fig. 1d). Moreover, ΔMZ B mice survived significantly longer than did MZ B-WT mice (Fig. 1e). Therefore, MZ B cells likely contribute to systemic inflammatory responses to LPS.

### MZ B cells produce pro-inflammatory cytokines

**Figure 2:**
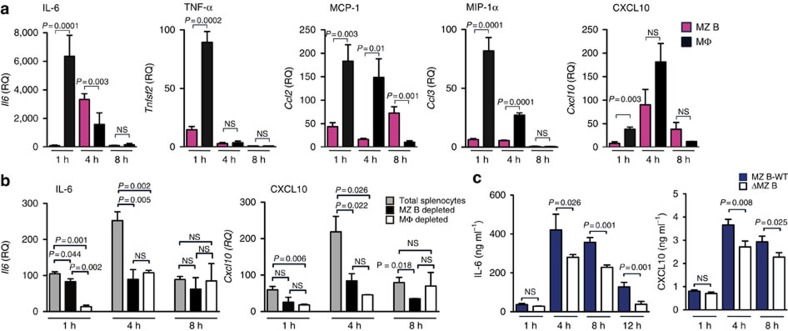
MZ B cells secrete cytokines and chemokines during endotoxic shock. (**a**,**b**) MZ B cells (MZ B) and macrophages (Mϕ) (**a**), or total splenocytes (grey) and splenocytes depleted of MZ B cells (black) or macrophages (white) (**b**) were purified from C57BL/6 mice after LPS injection at indicated time points after LPS injection and analysed for indicated cytokines and chemokines by quantitative RT–PCR. (**c**) Sera obtained from ΔMZ B mice at indicated time points after LPS injection were measured for IL-6 and CXCL10 by enzyme-linked immunosorbant assay. Data are representative of three independent experiments. Statistical analyses were performed with the unpaired Student's *t*-test. Error bars indicate s.d. NS, not significant.

To elucidate how MZ B cells are involved in systemic inflammatory responses, we examined whether MZ B cells produce pro-inflammatory cytokines or chemokines in response to i.v. injection of LPS. As expected, splenic macrophages quickly produced a large amount of various cytokines and chemokines after LPS injection (Fig. 2a). Unexpectedly, however, MZ B cells also produced a robust amount of IL-6 after LPS challenge. Notably, the relative expression of IL-6 by MZ B cells was significantly higher than that by macrophages 4 h after LPS injection (Fig. 2a). MZ B cells also produced chemokines, such as MCP-1 and CXCL10, but not tumour necrosis factor-α (TNF-α) or MIP-1α (Fig. 2a). We performed quantitative reverse transcription–PCR (RT–PCR) of total splenocytes and those depleted (by negative sorting) of either MZ B cells or macrophage populations; in this analysis, IL-6 and CXCL10 were produced primarily by macrophages at 1 h after LPS injection. However, MZ B cells and macrophages produced comparable amounts of both IL-6 and CXCL10 4 h after LPS injection (Fig. 2b). Furthermore, serum IL-6 and CXCL10 levels were significantly lower in ΔMZ B mice than MZ B-WT mice 4, 8 and 12 h after injection of LPS (Fig. 2c). Therefore, MZ B cells likely behave similarly as macrophages in inflammatory cascade by secreting pro-inflammatory cytokines and chemokines, such as IL-6 and CXCL10.

### IL-6 derived from MZ B cells is critical for endotoxic shock

**Figure 3:**
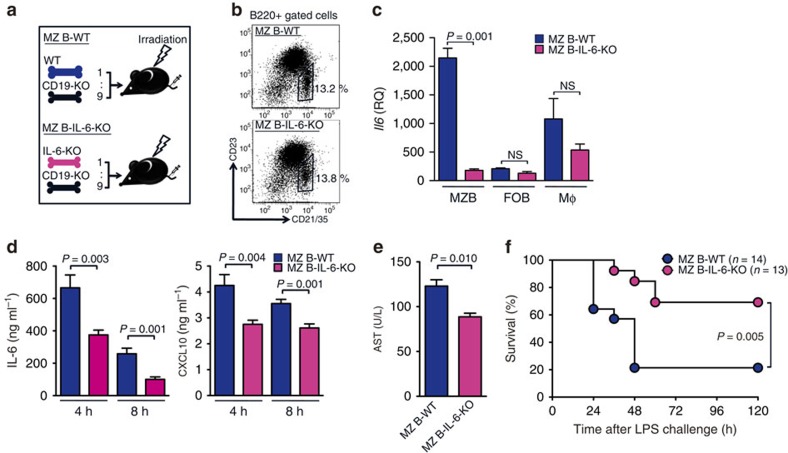
IL-6 from MZ B cells exacerbates systemic inflammatory responses against endotoxic shock. (**a**–**c**) Strategy to generate mice lacking IL-6 in MZ B cells (MZ B-IL6-KO) or control mice (MZ B-WT). Lethally irradiated mice received i.v. injection of mixed bone marrow cells from either WT or IL-6-deficient mice and CD19-deficient mice at a ratio indicated (**a**). Splenocytes were analysed for the proportion of MZ B cells among the B220^+^ cell population by flow cytometry (**b**). MZ B cells, follicular (FO) B cells and macrophages (Mϕ) were purified by flow cytometry 4 h after LPS injection and analysed for expression of *Il6* by quantitative RT–PCR (**c**). (**d**–**f**) MZ B-WT and MZ B-IL6-KO were analysed for serum levels of IL-6 and CXCL10 (**d**) at indicated time points and AST (**e**) 12 h after LPS injection and monitored for survival rate (**f**). Data are representative of at least two independent experiments. For mice survival, data are pooled of each experiment and the total numbers of mice are indicated. Statistical analyses were performed with the unpaired Student's *t*-test. The log-rank test was used for mice survival. Error bars indicate s.d. NS, not significant.

Since IL-6 seemed to be a dominant cytokine produced from MZ B cells, we investigated whether MZ B-cell-derived IL-6 is involved in systemic inflammatory responses to LPS. According to the approach described previously^24^, we generated mixed BM chimeric mice whose MZ B cells lacked IL-6 expression by transferring both *Il6*^−/−^ and *Cd19*^−/−^ BM cells at a ratio of 1:9, respectively, into lethally irradiated mice (MZ B-IL-6-KO; Fig. 3a). In MZ B-IL-6-KO mice, MZ B cells were derived from only *Il6*^−/−^ BM cells, whereas other blood cells developed from both *Il6*^−/−^ and *Cd19*^−/−^ BM cells at a ratio of 1:9, respectively. Flow cytometry analysis demonstrated that the development of MZ B cells derived from complemented BM cells were comparable between MZ B-WT and MZ B-IL-6-KO mice (Fig. 3b). The selective deletion of *Il6* transcripts in MZ B cells (but not in follicular (FO) B cells or macrophages) was confirmed after LPS injection into MZ B-IL6-KO mice (Fig. 3c). In response to LPS challenge, MZ B-IL-6-KO mice had significantly lower amounts of serum IL-6, CXCL10 and aspartate aminotransferase (AST) than did MZ B-WT mice (Fig. 3d,e). In addition, MZ B-IL-6-KO mice survived significantly longer compared with MZ B-WT mice (Fig. 3f). Therefore, IL-6 secreted by MZ B cells is critical in systemic inflammatory responses during LPS-induced endotoxic shock.

### Neutralization of IL-6 signalling attenuated endotoxic shock

**Figure 4:**
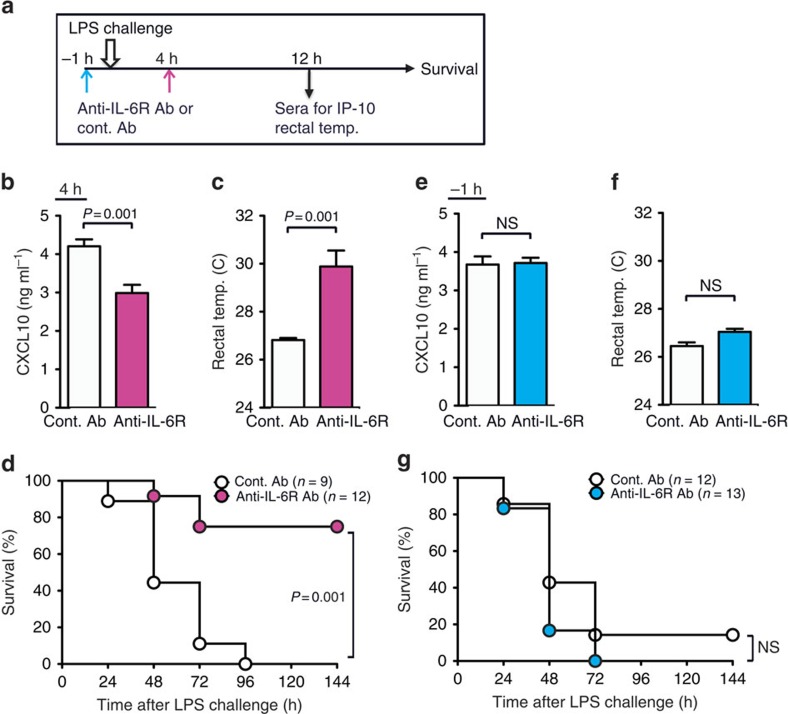
Neutralization of IL-6 by anti-IL-6R protects against endotoxic shock by LPS. (**a**) Experimental procedure of antibody injection to endotoxic shock. (**b**–**g**) Anti-IL-6R Ab or control Ab were i.v. injected to C57BL/6 mice 4 h after (**b**–**d**) or 1 h before LPS challenge (**e**–**g**). Mice were analysed for serum CXCL10 level (**b**,**e**) and rectal temperature (**c**,**f**) 12 h after LPS injection, and monitored for survival rate (**d**,**g**) after LPS injection. Data are representative of at least two independent experiments. For mice survival, data are pooled of each experiment and the total numbers of mice are indicated. Statistical analyses were performed with the unpaired Student's *t*-test. The log-rank test was used for mice survival. Error bars indicate s.d. NS, not significant. Ab, antibody; cont., control; temp., temperature.

We examined whether LPS-induced systemic inflammation was attenuated by neutralization of IL-6 signalling with an anti-IL-6 receptor (IL-6R) antibody^25^. To neutralize MZ B-cell-derived IL-6, mice received an i.v. injection of anti-IL-6R antibody (2 mg per mouse) 4 h after LPS injection (Fig. 4a). Mice treated with an anti-IL-6R antibody had significantly lower serum levels of IP-10 and higher rectal temperatures than did mice treated with a control antibody (Fig. 4b,c). Moreover, these mice survived significantly longer than did the control mice (Fig. 4d). However, treatment with this antibody 1 h before LPS injection did not change the serum levels of CXCL10, rectal temperature and survival of mice (Fig. 4e–g); consistently, IL-6 produced immediately after LPS injection suppressed TNF-α production, leading to exacerbation of systemic inflammatory responses^26^. These results are in agreement with the MZ B-cell production of IL-6 at 4 h, but not immediately, after LPS injection and with the attenuated inflammatory responses and prolonged survival of MZ B-IL6-KO mice.

### LPS directly stimulates MZ B cells via TLR4-coupled MyD88

**Figure 5:**
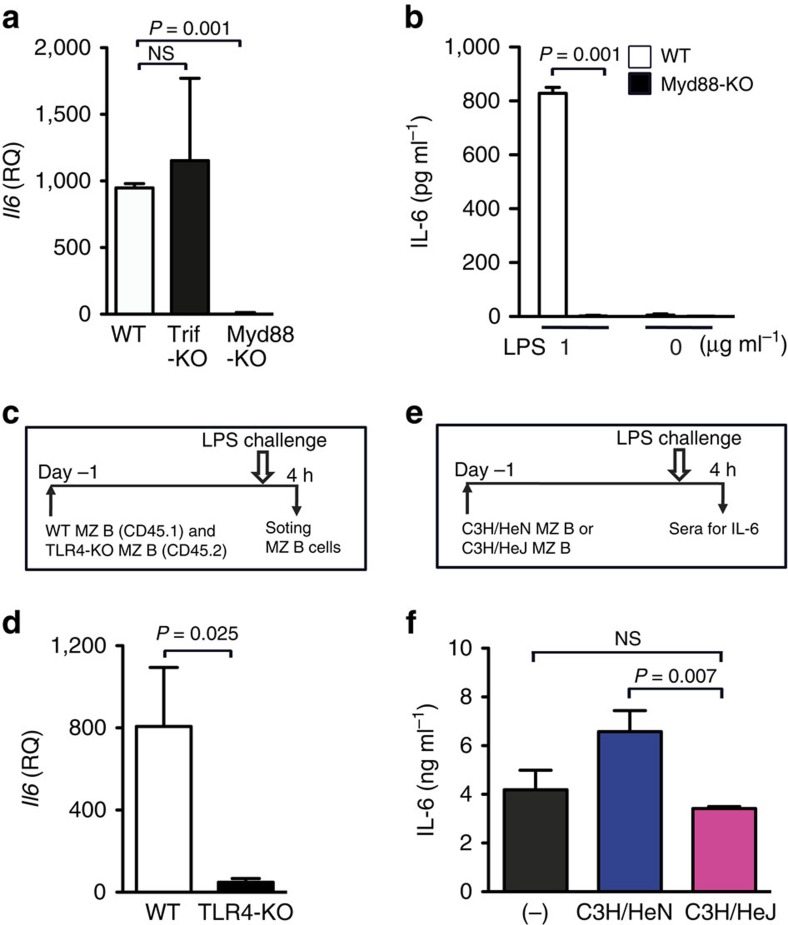
LPS directly stimulates MZ B cells for IL-6 production. (**a**) WT mice and TRIF or MyD88-deficient mice were i.v. injected with LPS. *Il6* expression in MZ B cells purified from indicated mice 4 h after LPS injection was determined by quantitative RT–PCR (qRT–PCR). (**b**) MZ B cells purified from WT or MyD88-deficient mice were cultured in the presence or absence of LPS for 24 h and then analysed for IL-6 production by ELISA. (**c**,**d**) WT mice (CD45.2) were transferred with WT (CD45.1) together with TLR-4-deficient (CD45.2) MZ B cells. Twenty-four hours later, mice were injected with LPS and then transferred WT (CD45.1) and TLR-4-deficient MZ B cells were purified by flow cytometry 4 h after LPS injection and analysed for expression of *Il6* by qRT–PCR. (**e**,**f**) C3H/HeJ mice were transferred with MZ B cells purified from C3H/HeJ or C3H/HeN mice. Twenty-four hours later, mice were injected with LPS and analysed for serum IL-6 levels 4 h after LPS injection by ELISA. Statistical analyses were performed with the unpaired Student's *t*-test. Error bars indicate s.d. NS, not significant.

To elucidate the signalling cascade for IL-6 production in MZ B cells during endotoxic shock, MZ B cells were purified from WT, *Myd*88^−/−^ or *Ticam*^−/−^ mice after LPS injection. *Il6* expression by *Ticam*^−/−^ and WT MZ B cells was comparable; however, *Myd*88^−/−^ MZ B cells had no detectable *Il6* transcripts (Fig. 5a). To examine whether LPS directly stimulates MZ B cells for IL-6 production, MZ B cells were purified from the spleens of WT and *Myd*88^−/−^ mice, stimulated with LPS and analysed for IL-6 production. In response to this LPS stimulation *in vitro*, WT MZ B cells produced IL-6; in contrast, *Myd*88^−/−^ MZ B cells did not (Fig. 5b). Moreover, MZ B cells were purified from the spleens of WT (CD45.1) or *Tlr4*^−/−^ (CD45.2) mice, labelled with carboxyl fluorescein succinimidyl ester (CFSE), and then transferred into WT mice (CD45.2). After stimulation with LPS, transferred MZ B cells were purified from the mice and analysed for *Il6* expression, demonstrating that *Il6* was detected in WT, but not *Tlr4*^−/−^, MZ B cells (Fig. 5c,d). These results formally provided the evidence that MZ B cells directly recognize LPS via TLR4 and produce IL-6. To further confirm this notion, MZ B cells were purified from the spleen of C3H/HeJ mice, which express mutated TLR4, or control C3H/HeN mice and transferred into C3H/HeJ mice. Then, mice were challenged with LPS and analysed for serum IL-6 levels. In contrast to C3H/HeJ mice that received MZ B cells derived from C3H/HeJ mice, mice that received MZ B cells derived from C3H/HeN mice showed significantly increased IL-6 levels in the sera (Fig. 5e,f). Taken together, these results indicated that LPS directly stimulates MZ B cells via TLR4-coupled MyD88 for IL-6 production *in vitro* and *in vivo*.

### Fcα/μR regulates IL-6 production from MZ B cells

**Figure 6:**
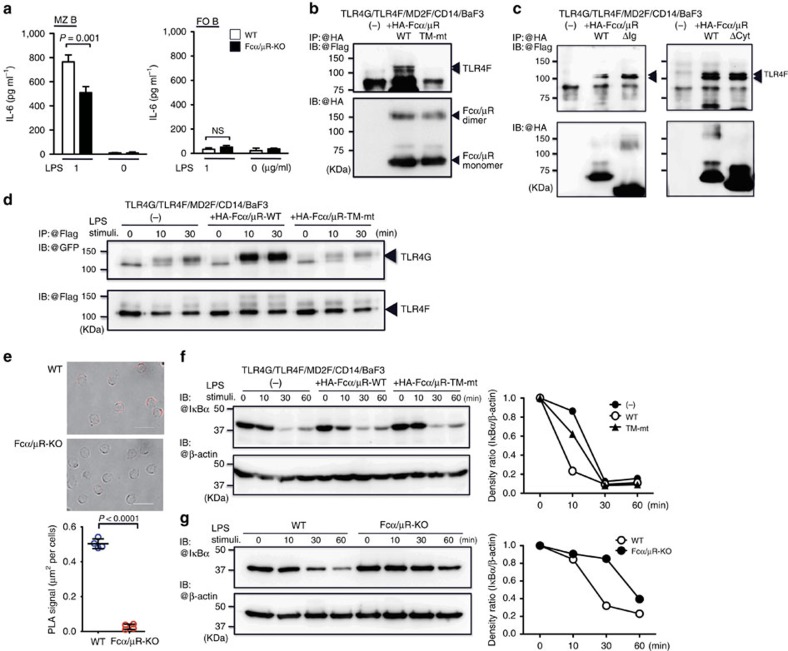
Fcα/μR associates with TLR4 and amplifies LPS-induced signalling cascade. (**a**) MZ B cells or FO B cells purified from WT or Fcα/μR-deficient mice were cultured in the presence or absence of LPS for 24 h and then analysed for IL-6 production by ELISA. (**b**,**c**) Ba/F3 cells stably expressing TLR4 fused with GFP (TLR4G), Flag-tagged TLR4 (TLR4F), Flag-tagged MD-2 (MD2F) and CD14 together with or without HA-tagged WT Fcα/μR (HA-Fcα/μR-WT) or Fcα/μR mutated at the transmembrane region (HA-Fcα/μR TM-mt) or lacking the immunoglobulin-like domain (HA-Fcα/μR ΔIg) or cytoplasmic portion (HA-Fcα/μR ΔCyt) were subjected to immunoprecipition with anti-HA, followed by immunoblotting with anti-Flag or anti-HA. (**d**,**f**) Ba/F3 cells stably expressing TLR4F, TLR4G, MD2F, CD14 or expressing these transduced proteins plus either HA-Fcα/μR-WT or HA-Fcα/μR TM-mt were stimulated or not with LPS and subjected to immunoprecipitation with anti-Flag (**d**) or not (**f**), followed by immunoblotting with anti-GFP (**d**), anti-Flag (**d**), anti-IκBα (**f**) or anti-β-actin (**f**). (**e**) MZ B cells were purified from WT or Fcα/μR-deficient mice and subjected to proximal ligation assay (PLA) to analyse association of Fcα/μR and TLR4. PLA signal was analysed by fluorescence microscopy. (**g**) MZ B cells purified from WT or Fcα/μR-deficient mice were stimulated or not with LPS and subjected to immunoblotting with anti-IκBα or anti-β-actin. The graph shows the kinetics of densities of each band. Data are representative of three independent experiments. Statistical analyses were performed with the unpaired Student's *t*-test. Error bars indicate s.d. IB, immunoblotting; IP, immunoprecipitation; NS, not significant.

To further analyse this signalling pathway for IL-6 production in MZ B cells, we focused on Fcα/μR (CD351) (refs 15, 16), a cell surface molecule that is highly expressed on MZ B cells^15^,^27^ (Supplementary Fig. 1). We observed that MZ B cells from Fcα/μR-deficient mice had significantly impaired IL-6 production after *in vitro* and *in vivo* stimulations with LPS (Fig. 6a; Supplementary Fig. 2). In contrast, both WT and Fcα/μR-deficient FO B cells produced significantly less amount of IL-6 compared with MZ B cells after stimulation with LPS *in vitro* (Fig. 6a). The physical association of Fcα/μR with TLR4 was indicated by the co-immunoprecipitation analysis of a Ba/F3-transfected cell line stably expressing haemagglutinin (HA)-tagged Fcα/μR, Flag-tagged TLR4, GFP-fused TLR4, Flag-tagged MD2 and CD14 (Fig. 6b). This association of Fcα/μR with TLR4 was not altered after LPS stimulation (Supplementary Fig. 3A). In contrast, there was no co-immunoprecipitation with TLR4 from Ba/F3 cells expressing HA-tagged, mutated Fcα/μR (TM-mt), whose transmembrane region was substituted with that of human allergin S2 (refs 28, 29; Fig. 6b; Supplementary Fig. 3B). However, Fcα/μR was co-immunoprecipitated with TLR4 when the extracellular Ig domain or cytoplasmic region of Fcα/μR was deleted (Fig. 6c; Supplementary Fig. 3B); Fcα/μR likely requires the transmembrane region for association with TLR4. In BaF3 cells stably expressing TLR4 components, GFP-fused TLR4 is co-immunoprecipitated with Flag-tagged TLR4 as a result of LPS-induced TLR4 oligomerization^30^,^31^. We observed that LPS-induced TLR4 oligomerization was enhanced in cells stably expressing WT Fcα/μR; however, it was not seen in cells expressing mutated Fcα/μR (TM-mt) (Fig. 6d). Therefore, Fcα/μR may enhance LPS-induced TLR4 oligomerization. We also found the physical association of TLR4 with Fcα/μR in primary MZ B cells by *in situ* proximity ligation assay (PLA; Fig. 6e). Next, we investigated whether Fcα/μR has an effect on NF-κB signalling. The TLR4-mediated NF-κB signalling cascade results in IκBα degradation^30^,^31^. LPS-induced IκBα degradation was enhanced in cells expressing WT Fcα/μR but not mutated Fcα/μR (TM-mt) (Fig. 6f). In addition, after LPS stimulation, Fcα/μR-deficient MZ B cells had defective IκBα degradation compared with WT MZ B cells (Fig. 6g). Therefore, Fcα/μR may enhance NF-κB signalling. However, we observed that TLR4 oligomerization and NF-κB signalling after LPS stimulation were comparable between BaF3 cells expressing WT Fcα/μR and mutated Fcα/μR lacking cytoplasmic region (ΔCyt; Supplementary Fig. 4), suggesting that Fcα/μR-mediated signalling is not required for the enhanced NF-κB signalling. We also observed that NF-κB signalling was not changed in BaF3 transfectant expressing Fcα/μR after LPS stimulation even under culture without the ligand for Fcα/μR (that is, IgA and IgM) using serum from Jh-KO mice (Supplementary Fig. 5). In addition, Fcα/μR-mediated enhancement of IL-6 production from MZ B cells did not require IgM *in vivo* (Supplementary Fig. 2). These results indicate that Fcα/μR did not require the ligands in the serum for the enhancement of LPS-induced IL-6 production in MZ B cells.

### Fcα/μR on MZ B cells regulates systemic inflammation

**Figure 7:**
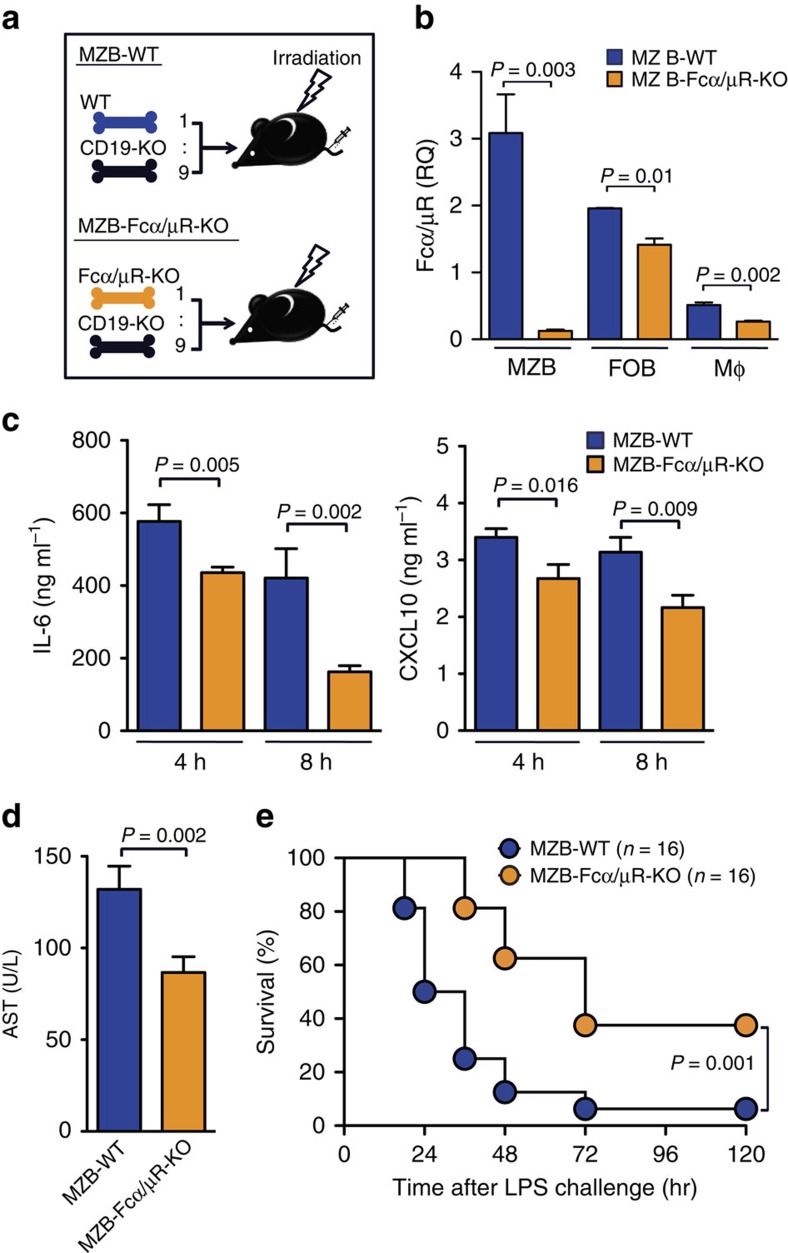
Fcα/μR on MZ B cell amplifies systemic inflammatory responses. (**a**,**b**) Strategy to generate mice lacking Fcα/μR in MZ B cells (MZ B-Fcα/μR-KO) or control mice (MZ B-WT). Lethally irradiated mice received i.v. injections of mixed bone marrow cells from either WT or Fcα/μR-deficient mice and CD19-deficient mice at a ratio indicated (**a**). MZ B cells, FO B cells and macrophages (Mϕ) purified from MZ B-WT or MZ B-Fcα/μR-KO mice by flow cytometry were analysed for the expression of *Fcamr* by quantitative RT–PCR (**b**). (**c**–**e**) MZ B-WT or MZ B-Fcα/μR-KO mice were injected with LPS and then analysed for serum levels of IL-6 and CXCL10 4 and 8 h after LPS injection (**c**), AST 12 h after LPS injection (**d**) and monitored for survival rate (**e**). Data are representative of at least two independent experiments. For mice survival, data are pooled of each experiment and the total numbers of mice are indicated. Statistical analyses were performed with the unpaired Student's *t*-test. The log-rank test was used for mice survival. Error bars indicate s.d. NS, not significant.

We analysed the importance of Fcα/μR expressed on MZ B cells in systemic inflammatory responses to LPS *in vivo*; we established MZ B-cell-specific Fcα/μR-deficient mice (MZ B-Fcα/μR-KO) by transferring BM cells from Fcα/μR-deficient and *Cd19*^*−/−*^ mice at a ratio of 1:9, respectively, into lethally irradiated mice (Fig. 7a). In MZ B-Fcα/μR-KO mice, Fcα/μR was selectively deleted in MZ B cells (Fig. 7b). After LPS injection, MZ B-Fcα/μR-KO mice had significantly lower levels of serum IL-6, CXCL10 and AST than did MZ B-WT mice (Fig. 7c,d). Moreover, after LPS injection, MZ B-Fcα/μR-KO mice survived significantly longer than MZ B-WT mice (Fig. 7e). Taken together, these findings indicate that Fcα/μR plays an important role in inflammatory responses to LPS by augmenting TLR4-mediated signalling in MZ B cells.

### Anti-IL-6 antibody attenuates sepsis induced by *E. coli*

**Figure 8:**
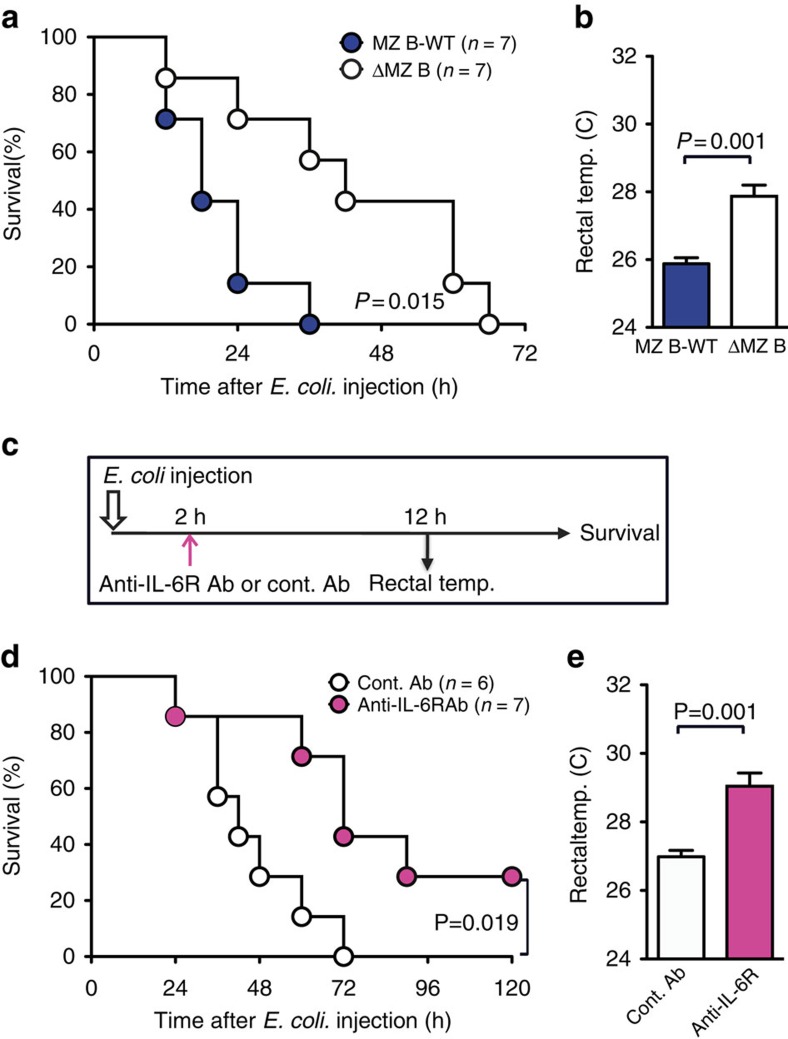
Neutralization of IL-6 by anti-IL-6R protects against sepsis by *E. coli* injection. (**a**,**b**) Mice lacking MZ B cells (ΔMZ B mice) or control mice (MZ B-WT) were injected with *E. coli* and monitored for survival rate (**a**) and analysed for rectal temperature 12 h after *E. coli* injection (**b**). (**c**–**e**) Anti-IL-6R Ab or control Ab were i.v. injected to the mice 2 h after *E. coli* injection (**c**) and monitored for survival rate (**d**) and analysed for rectal temperature (**e**) 12 h after LPS injection. Data are pooled of each experiment and the total numbers of mice are indicated. Statistical analyses were performed with the unpaired Student's *t*-test. The log-rank test was used for mice survival. Error bars indicate s.d. Ab, antibody; cont., control; NS, not significant; temp., temperature.

To analyse the role of MZ B cells and IL-6 in a more pathophysiological relevant sepsis model, we injected i.v. *E. coli*. ΔMZ B mice showed significantly longer survival and milder decrease in the rectal temperature than did MZ B-WT mice after administration of *E. coli* (Fig. 8a,b). In addition, treatment of mice with anti-IL-6R antibody 2 h after *E. coli* injection significantly prolonged the survival and showed milder decrease in the rectal temperature compared with mice that treated with control antibody (Fig. 8c–e). We also examined the effect of anti-IL-6R antibody on the survival of mice after caecum ligation and puncture (CLP), a widely used sepsis model^32^. Since mice after CLP showed delayed IL-6 responses compared with those after LPS or *E. coli* injection (Supplementary Fig. 6A), we injected mice with anti-IL-6R antibody 6–8 h after CLP to neutralize the late phase of IL-6. Mice treated with anti-IL-6R antibody showed prolonged survival and milder decrease in the rectal temperature compared with mice treated with control antibody (Supplementary Fig. 6B,C). Together, these results indicated the critical role of MZ B cells and IL-6 for the exacerbation of sepsis induced by *E. coli* injection and CLP.

## Discussion

MZ B cells have been recognized as antibody producing cells against blood-borne pathogens^7^,^8^. In the present study, we showed that MZ B cells produced a significant amount of inflammatory cytokines and chemokines in response to LPS stimulation. Moreover, by establishing mixed BM chimeric mice lacking MZ B cells, we demonstrated the critical role of MZ B cells for systemic inflammatory responses during endotoxic shock. Of note, IL-6 produced by MZ B cells played a pivotal role in exacerbation of endotoxic shock, as revealed by the analyses of IL-6-deficient mice specifically in MZ B cells.

Using sepsis models, we investigated the role of IL-6 in systemic inflammatory responses. Although IL-6 is a pro-inflammatory cytokine that exacerbates acute and chronic phases of inflammation, Xing *et al*.^26^ previously demonstrated that IL-6-deficient mice showed significantly shorter survival than did WT mice after LPS injection. They reported that IL-6 played as an anti-inflammatory cytokine that suppressed the production of pro-inflammatory cytokines such as TNF-α in the very early phase after LPS injection, leading to the attenuation of systemic inflammatory responses^26^. In the present study, we showed that neutralization of IL-6R signalling by a neutralizing anti-IL-6R antibody at the time points around (1 h before) LPS challenge did not show any effect on the survival of mice. Our results together with previous reports suggest that IL-6, which is mainly derived from macrophages, at the very early phase of inflammatory response to endotoxin may not augment systemic inflammation. However, we showed that a significant amount of IL-6 was produced by MZ B cells as well as by macrophages at 4 h after LPS challenge. Neutralization of IL-6R signalling around at this time point (2–4 h after LPS or *E. coli* injection) significantly prolonged survival of mice after LPS or *E. coli* injection, indicating that IL-6 produced at delayed time points a few hours after exposure of endotoxin indeed exacerbates systemic inflammation. In accordance with this idea, treatment of mice with anti-IL-6R antibody at the late phase (6–8 h) of CLP prolonged the survival of mice compared with treatment with control antibody. These results suggest that timely neutralization of IL-6R-mediated signalling may be useful for the treatment of sepsis.

We observed that IL-6 production from MZ B cells in response to LPS required Fcα/μR even in the absence of its ligands (IgA or IgM) in the serum. Since MZ B cells harbour BCR reactive to LPS^33^, we speculated that membrane IgM or IgM quickly produced in response to LPS from MZ B cells forms a complex with LPS, which also interacts with Fcα/μR as well. Since Fcα/μR associates with TLR4 via its transmembrane region, interaction of Fcα/μR with IgM-coated LPS may enhance LPS-induced oligomerization of TLR4, leading to the amplification of MZ B-cell activation. Similar mechanism was previously reported with a C-type lectin SIGNR1 (CD209b), a capturing receptor for *E. coli*^34^,^35^. On binding to *E. coli*, SIGNR1 enhances TLR4 oligomerization via association with TLR4, and increases cytokine production from macrophages^31^. Further analysis should be required to clarify how Fcα/μR is involved in the amplification of TLR4 signalling.

The involvement of B cells in inflammatory responses has been demonstrated in several disease models. In a peritonitis model induced by CLP, B cells produce CXCL10 in response to type I interferon secreted during peritonitis and amplifies the inflammatory responses, leading to efficient bacterial eradication^36^. During myocardiac infarction induced by coronary artery ligation, B cells produced CXCL7, which recruits inflammatory monocyte to the heart and impairs myocardium remodelling and function^37^. Recent studies have identified a novel B-cell subset, named innate response activator B cells, differentiated from B1 B cells in the peritoneal cavity during CLP-induced peritonitis. Innate response activator B cells secret granulocyte–macrophage colony-stimulating factor for protection from bacterial infections^38^. Ping *et al*. reported IL-35-producing B cells with CD138^high^ plasma cells phonotype, which suppress experimental autoimmune myelitis and host defence against *Salmonella enterica* infection^39^. In addition, Tedder's group had identified regulatory B-cell population producing IL-10, named B10 cells^40^,^41^. B10 cells expand during experimental arthritis^42^ and *Listeria monocytogenes* infection^43^, leading to the suppression of T cells responses. Thus, various B-cell subsets exist and control inflammatory responses via secreting pro- or anti-inflammatory cytokines and chemokines.

Among B-cell population, MZ B cells are primarily recognized as quickly antibody producing cells, critical for the early immune defences against blood-borne pathogens^7^,^8^. It was reported that MZ B cells secret an anti-inflammatory cytokine IL-10 after *Listeria monocytogenes* infection^44^. Indeed, precursor cells for B10 cells (B10pro) are recently identified within MZ B cells population^45^. In contrast, our current study has unveiled a pro-inflammatory role of MZ B cells: the production of IL-6 that is responsible for LPS-mediated endotoxic shock. Thus, MZ B cells are not only just antibody producer but also regulator for immune responses. In humans, IgM^+^ IgD^+^ CD27^+^ B cells were identified as a counterpart of rodent MZ B cells^46^,^47^. They are present in the blood as well as in the spleen^48^. However, the functional characteristics of human MZ B cells have remained unclear. Future studies are required for elucidation of the functional role of human MZ B cells in inflammatory responses.

## Methods

### Mice

C57BL/6J, C3H/HeJ and C3H/HeN mice were purchased from Clea Japan (Tokyo, Japan). The genetic background of the genetically engineered mice used was C57BL/6J. *Il6*^*−/−*^ (IL-6-KO), *Cd19*-Cre, *Jh*^−/−^ (Jh-KO) and C57BL/6-Ly5.1 mice were purchased from Jackson Laboratory (Bar Harbor, ME, USA); mice with homozygous *Cd19* deficiency (*Cd19*^cre/cre^) were used as *Cd19*^*−/−*^ mice. *Myd88*^*−/−*^ (Myd88-KO), *Ticam*^*−/−*^ (Trif-KO) and *Tlr4*^*−/−*^ (TLR4-KO) mice were purchased from Oriental BioService (Kyoto, Japan). Fcα/μR^*−/−*^ (Fcα/μR-KO) mice were generated in our laboratory, as previously described, and backcrossed onto the C57BL/6 genetic background for 12 generations. Only female mice between the ages of 8 weeks and 12 weeks were used for the experiments. All experiments were performed in accordance with the guidelines of the animal ethics committee of the University of Tsukuba Animal Research Center.

### Generation of BM chimeric mice

Lethally irradiated (9 Gy) C57BL/6 mice received i.v. injections of 5 × 10^6^ BM cells total (mixture of indicated populations). For establishing ΔMZ B mice, BM cells from *Cd19*^*−/−*^ mice were injected into lethally irradiated C57BL/6 mice. For establishing MZ B-cell-specific gene-targeting mice, BM cells from *Cd19*^*−/−*^ mice were mixed with BM cells from *Il6*^*−/−*^ or Fcα/μR^*−/−*^ mice at a 9:1 ratio, respectively. These cells were then injected into lethally irradiated C57BL/6 mice. Eight weeks after the transfer, mice were used for experiments.

### Experimental sepsis

WT or BM chimeric mice received i.v. injection of LPS (600 μg per mouse) from *E. coli* (O55:B5; Sigma-Aldrich, St Louis, MO, USA) or *E. coli* (1.5 × 10^9^ CFU per mouse; DH10B). CLP were performed as described previously^32^.The caecum was exposed by a 1–2-cm midline incision in the ventral abdomen, ligated at ∼12 mm from its distal portion, and punctured twice with a 23-G needle in the ligated segment. The abdomen was closed in two layers, and 1 ml of sterile saline was administered subcutaneously. Serum levels of inflammatory cytokines and chemokines 1, 4, 8 or 12 h after CLP were measured and mortality of mice was monitored. AST values in the serum were measured using a Fuji DRI-CHEM 3,500-V slide analyser (Fujifilm, Japan).

### Antibodies

Anti-mouse CD3ɛ (145-2C11), CD4 (RM4-5), CD5 (53-7.3), CD8a (53-6.7), CD11b (M1/70), CD11c (HL3), CD21/35 (7G6), CD23 (B3B4), CD45.1 (A20), CD45.2 (104), Ly6C (AL-21), Ly6G (1A8), B220 (RA3-6B2) and NK1.1 (PK136), and IgM (R6-60.2) monoclonal antibodies and isotype-matched control antibodies were purchased from BD Biosciences (San Jose, CA, USA) and used for staining following cell populations (MZ B cells (B220^+^ CD21/35^high^ CD23^−^), follicular B cells (B220^+^ CD21/35^+^ CD23^+^), macrophages (CD11b^+^ Ly6G^−^ NK1.1^−^), CD4 T cells (CD4^+^ CD3^+^), CD8 T cells (CD8a^+^ CD3^+^), natural killer cells (CD11b^+^ Ly6G^−^ NK1.1^+^), neutrophils (CD11b^+^ Ly6G^+^), dendritic cells (CD11c^+^ B220^−^), plasmacytoid dendritic cells (CD11c^+^ B220^+^), immature/mature B cells (B220^+^ IgM^+^), inflammatory monocytes (CD11b^+^ Ly6C^high^), B1a B cells (B220^+^ CD5^+^) and B2/B1b B cells (B220^+^ CD5^−^)). Monoclonal antibodies against HA (3F10) was purchased from Roche (Penzberg, Germany). Anti-GFP polyclonal antibody was purchased from Life Technologies (Carlsbad, CA, USA). Anti-IκBα polyclonal antibody was purchased from Cell Signaling (Danvers, MA, USA). Anti-mouse β-actin (AC15) and Flag (M2) monoclonal antibody and anti-Flag polyclonal antibody were purchased from Sigma-Aldrich. Mouse IgG1-chimeric anti-IL-6R antibody (MR16-1)^25^ was kindly provided by Tadamitsu Kishimoto and Chugai Pharmaceuticals (Shizuoka, Japan). Anti-Fcα/μR monoclonal antibody, TX57 and TX61 were generated, as described. Where indicated, TX25 (mouse IgG1) was used as a control antibody. The amount of antibodies used for flow cytometry analyses was 50 μl (20–25 μg ml^−1^) per 1 × 10^6^ cells.

### Generation of stable cell lines

The mouse pro-B-cell line Ba/F3 stably expressing Flag-tagged TLR4, TLR4 fused with GFP, Flag-tagged MD2 and CD14, as described previously^30^, was maintained in RPMI 1640 containing 10% fetal calf serum, 2 mM L-glutamine, 100 U ml^−1^ penicillin, 100 μg ml^−1^ streptomycin and recombinant murine IL-3 (∼70 U ml^−1^). The source of recombinant murine IL-3 was medium conditioned by Chinese hamster ovary cells that had been genetically engineered to produce murine IL-3 up to ∼70,000 U ml^−1^ (ref. 30). WT Fcα/μR or three Fcα/μR mutants (lacking the Ig domain (ΔIg), lacking the cytoplasmic portion (ΔCyt) or substituting the transmembrane region with that of human allergin S2 (TM-mt)) were tagged with HA at the N terminus then subcloned into a pMX retrovirus vector. Constructed pMX vectors were used for establishing Ba/F3 cells stably expressing Flag-TLR4, TLR4-GFP, Flag-MD-2 and CD14 with WT or mutant Fcα/μR, as previously described^30^.

### Enzyme-linked immunosorbant assay

The concentrations of IL-6 and IgM in serum or culture supernatant were measured by enzyme-linked immunosorbant assay. Anti-mouse IL-6 (MP5-20F3) and mouse IgM (II/41) were used as capture antibodies. Biotinylated anti-mouse IL-6 (MP5-32C11) or horseradish peroxidase-conjugated anti-mouse IgM polyclonal antibody was used as the detection antibody. Serum CXCL10 concentration was measured using a mouse CXCL10 Platinum ELISA kit (eBioscience, San Diego, CA, USA). Inflammatory cytokine/chemokine production in mice sera were also measured using cytokine bead array (CBA; BD Biosciences) where indicated.

### CBA analysis

The concentrations of multiple inflammatory cytokines and chemokines were measured using CBA analysis (BD Biosciences) where indicated, according to the manufacturer's instructions.

### Quantitative RT–PCR

Total RNA was isolated from cell pellets using Isogen (Nippon Gene, Tokyo, Japan) and then used for reverse trancription using a High-Capacity cDNA RT kit (Applied Biosystems, Carlsbad, CA, USA). Real-time PCR analysis of *Fcamr, Il6, Tnsfs2, Ccl2, Ccl3, Cxcl10* and *Actb* (β-actin) was performed using an ABI 7500 sequence detector (Applied Biosystems) with Power SYBR Green PCR Master Mix (Applied Biosystems). The primers were as follows: *Fcamr* forward: 5′-ctccctttcaggtacaaatgca-3′ and *Fcamr* reverse: 5′-tctttgatgcctgttgactgag-3′; *Il6* forward: 5′-gaggataccactcccaacagacc-3′ and *Il6* reverse: 5′-aagtgcatcatcgttgttcataca-3′ (for IL-6-KO mice, *Il6* forward: 5′-agttgccttcttgggactga-3′ and *Il6* reverse: 5′-tccacgatttcccagagaac-3′); *Tnsfs2* forward: 5′-gggccaccacgctgttc-3′ and *Tnsfs2* reverse: 5′-ggtctgggccatagaactgatg-3′; *Ccl2* forward: 5′-ttaaaaacctggatcggaaccaa-3′ and *Ccl2* reverse: 5′-gcattagcttcagatttacgggt-3′; *Ccl3* forward: 5′-ccaagtcttctcagcgccat-3′ and *Ccl3* reverse: 5′-tccggctgtaggagaagcag-3′; and *Cxcl10* forward: 5′-cccacgtgttgagatcattgc-3′ and *Cxcl10* reverse: 5′-gaggctctctgctgtccatc-3′. The *Actb* level was measured as an internal control to normalize the data (forward primer: 5′-actgtcgagtcgcgtcca-3′ and reverse primer: 5′-gcagcgatatcgtcatccat-3′). The messenger RNA level was determined relative to that in the spleen. All values were determined in triplicate.

### Isolation and *in vitro* stimulation of MZ and FO B cells

Naive MZ B cells and FO B cells were sorted on the gates of B220^+^ CD21/35^high^ CD23^−^ and B220^+^ CD21/35^+^ CD23^+^ cells, respectively, from the spleens using flow cytometry (FACSAria, BD Biosciences). MZ B cells from the spleen of mice after LPS injection were sorted on the gate of B220^+^ CD23^−^ CD1d^high^ cells. Purified MZ B cells were cultured in 96-well plates with 1 μg ml^−1^ LPS for 24 h, and measured for IL-6 production. For analysis of IκBα degradation, purified MZ B cells were stimulated with 1 μg ml^−1^ LPS and analysed by immunoblotting.

### Immunoblot analysis

For analysis of the association between TLR4 and Fcα/μR, BaF3 transfectants were lysed in buffer containing 1% digitonin, 0.12% Triton X-100, 150 mM NaCl, 20 mM triethanolamine and protease inhibitors (1 mM phenylmethylsulfonyl fluoride and 10 U ml^−1^ aprotinin). The lysates were immunoprecipitated with anti-Flag monoclonal antibody, separated by SDS–polyacrylamide gel electrophoresis (SDS–PAGE) under reducing conditions, and then immunoblotted with anti-HA monoclonal antibody or anti-Flag polyclonal antibody. For analysis of TLR4 oligomerization, cell lysates of BaF3 transfectants were stimulated with 1 μg ml^−1^ LPS for 10 or 30 min, and then lysed in buffer containing 50 mM Tris-HCl (pH 7.6), 150 mM NaCl, 25 mM CaCl_2_, 0.5% Triton X-100 and protease inhibitors. The lysates were immunoprecipitated with anti-Flag monoclonal antibody, separated by SDS–PAGE under reducing conditions and then immunoblotted with anti-GFP (Life Technologies) or anti-Flag polyclonal antibodies (Sigma-Aldrich). For analysis of IκBα degradation, purified MZ B cells or BaF3 transfectants were stimulated with 1 μg ml^−1^ LPS for 10, 30 or 60 min, and then lysed in buffer containing 1% NP-40, 0.12% Triton X, 150 mM NaCl and protease inhibitors. Total cell lysates were separated by SDS–PAGE under reducing conditions and immunoblotted with anti-IκBα polyclonal antibody (Cell Signaling). Images have been cropped for presentation. Full-size images are presented in Supplementary Fig. 7.

### MZ B cells transfer experiment

MZ B cells (1–5 × 10^6^) from the spleen of C57BL/6-Ly5.1 (CD45.1^+^), TLR4-KO (CD45.2^+^) or Fcα/μR-KO (CD45.2^+^) mice were purified, labelled with CFSE and transferred i.v. into recipient mice. Next day, CFSE^+^ CD45.1^+^ (WT) and CFSE^+^ CD45.2^+^ (TLR4-KO or Fcα/μR-KO) MZ B cells in the spleen were sorted using flow cytometry (FACSAria, BD Biosciences) 4 h after LPS injection (600 μg per mouse) for analysis of *Il6* transcript expression with quantitative RT–PCR. In some experiment using Jh-KO recipient mice, 500 μl of PBS or C57BL/6 mice serum was injected (100 μl and 400 μl via i.v. and intraperitoneally, respectively) to those mice 0.5 h before LPS challenge.

Where indicated, 1–5 × 10^6^ MZ B cells from C3H/HeJ and C3H/HeN were transferred into C3H/HeJ mice, and then challenged with LPS next day. IL-6 levels in sera were measured 4 h after LPS challenge.

### Proximity ligation assay

MZ B cells purified from the spleen of WT and Fcα/μR-KO mice by flow cytometry were fixed with aceton and incubated with mouse anti-mouse Fcα/μR monoclonal antibody (TX57) together with rabbit anti-mouse TLR4 monoclonal antibody (ab13556, Abcam). DsRed PLA signals were developed using anti-mouse PLUS and anti-rabbit MINUS PLA probes using Duolink in situ PLA kit (Olink Bioscience), according to the manufacturer's instructions. Cells were analysed by fluorescence microscopy (BZ-X710, Keyence) using BZ-X analyser software. Fluorescent signals of PLA were measured and calculated per cell.

### Statistics

Statistical analyses were performed with the unpaired Student's *t*-test. The log-rank test was used for mice survival. *P* values<0.05 were considered statistically significant.

## Additional information

**How to cite this article:** Honda, S.-i. *et al*. Marginal zone B cells exacerbate endotoxic shock via interleukin-6 secretion induced by Fcα/μR-coupled TLR4 signalling. *Nat. Commun.* 7:11498 doi: 10.1038/ncomms11498 (2016).

## Supplementary Material

Supplementary Information Supplementary Figures 1-7 and Supplementary Table 1
