# Supplementary material for: Marginal zone B cells exacerbate endotoxic shock via interleukin-6 secretion induced by Fcα/μR-coupled TLR4 signalling
Source: Nat Commun. 2016 May 5;7:11498. doi: 10.1038/ncomms11498 (PMC4858745; doi:10.1038/ncomms11498)
Supplement: Supplementary Information — Supplementary Figures 1-7 and Supplementary Table 1 [file ncomms11498-s1.pdf]

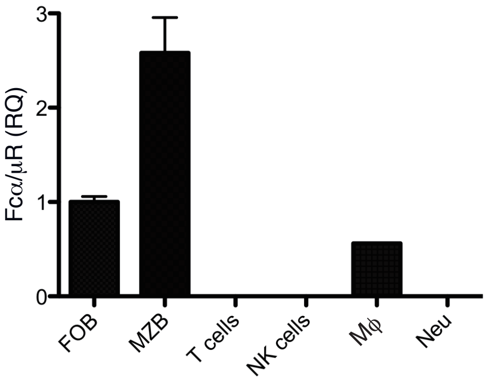

Supplementary Figure 1. Fcα/μR expression in splenocytes.

Follicular B cells (FOB), marginal zone B cells (MZB), T cells, natural killer cells (NK cells), macrophages (Mϕ), and neutrophils (Neu) were purified from spleen of naive C57BL/6 mice and analyzed for expression of *Fcamr* by quantitative RT-PCR.

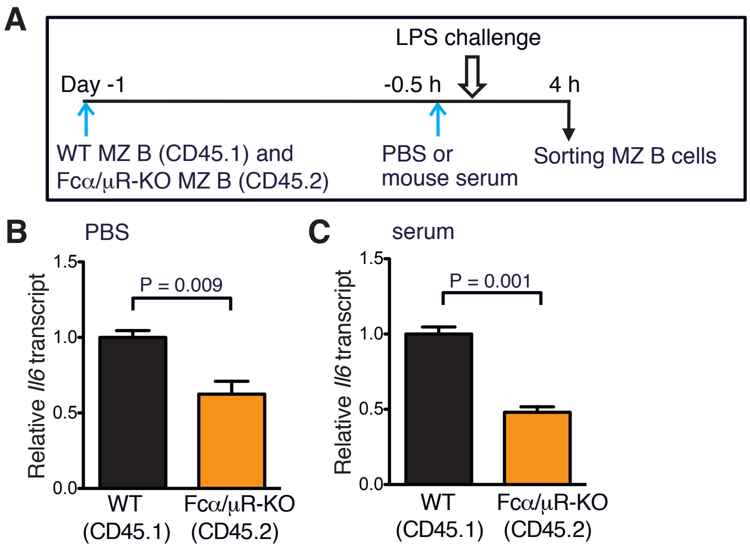

Supplementary Figure 2. Fcα/μR does not require ligand binding for enhancement of IL-6 production from MZ B cells *in vivo* .

(**A**) Experimental procedures for transfer of MZ B cells. WT (CD45.1) and Fcα/μR-KO (CD45.2) MZ B cells were labeled with CFSE and transferred into antibody-deficient Jh-KO mice. One day later, PBS (**B**) or the serum from C57BL/6 mouse (**C**) was injected 0.5 h before LPS challenge. WT and Fcα/μR-KO MZ B cells were sorted from the spleen 4 h after LPS challenge and analyzed for *Il6* transcripts by quantitative RT-PCR. The relative *Il6* transcripts to WT MZ B cells are shown, respectively.

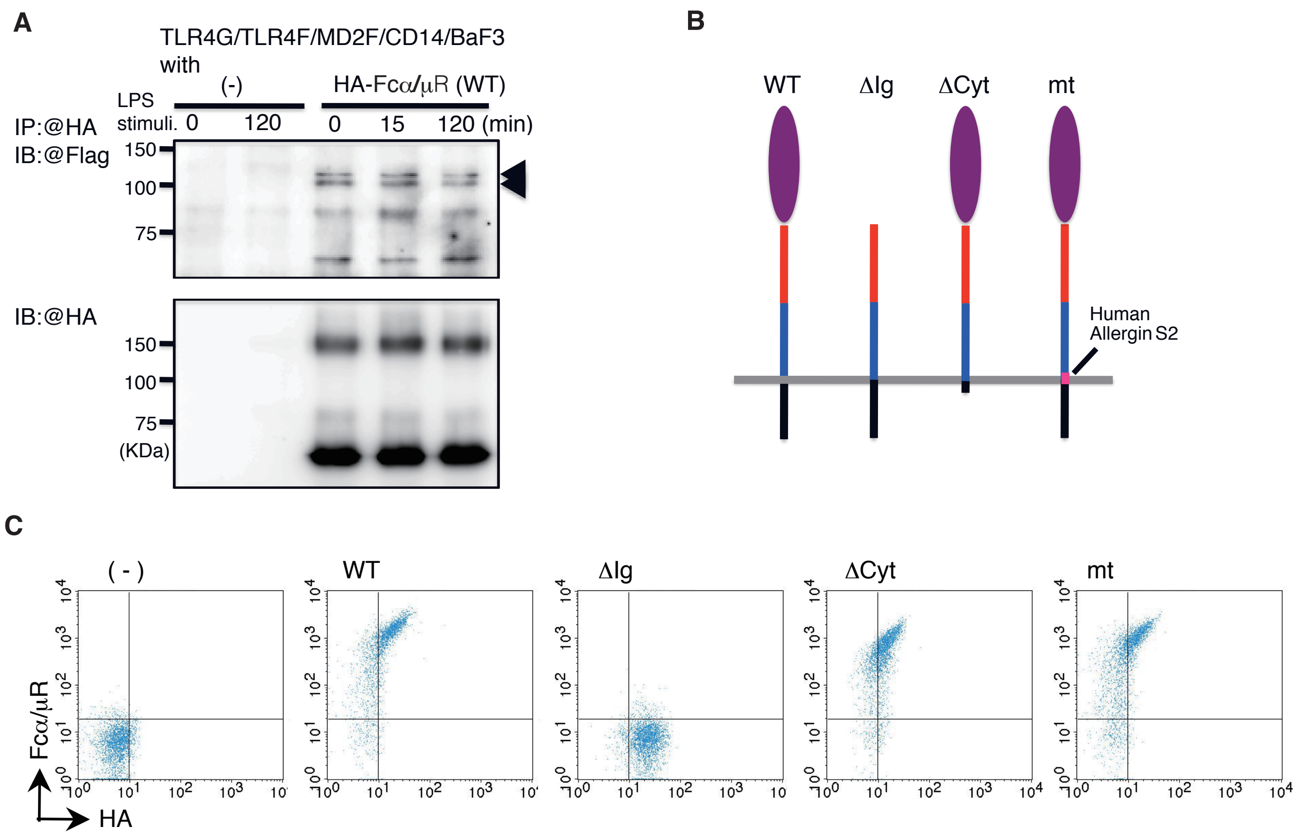

Supplementary Figure 3. Fcα/μR associates with TLR4 at steady state via neither Ig domain or cytoplasmic region.

(**A**) Immunoprecipitation with anti-HA mAb of Ba/F3 cells stably expressing Flag-tagged TLR4 (Flag-TLR4), TLR4 fused with GFP (TLR4-GFP), Flag-tagged MD-2 (Flag-MD2), and CD14 [(-)] together with or without HA-tagged Fcα/μR (WT). Immunoblotting was performed with anti-Flag pAb or anti-HA mAb, as indicated. ▲, Flag-TLR4. (**B**) Schematic presentation of Fcα/μR (WT) and the Fcα/μR mutants used for transfection: lacking Ig domain (ΔIg), lacking cytoplasmic region (ΔCyt), or substituting its transmembrane region with that of human Allergin S2 (mt). (**C**) Flow cytometry staining [anti-Fcα/μR mAb (TX61) and anti-HA mAb (3F10)] of Ba/F3 cells stably expressing Flag-TLR4, TLR4-GFP, Flag-MD-2, CD14 [(-)]

together with or without HA-tagged Fcα/μR (WT) and its mutants (ΔIg, ΔCyt, and mt). (**C**) Immunoprecipitation with anti-HA mAb of Ba/F3 cells stably expressing Flag-TLR4, TLR4-GFP, Flag-MD-2, CD14 [(-)] together with or without Fcα/μR (WT), Fcα/μR (ΔIg), or Fcα/μR (ΔCyt). Immunoblotting was performed with anti-Flag pAb or anti-HA mAb, as indicated. ▲, Flag-TLR4.

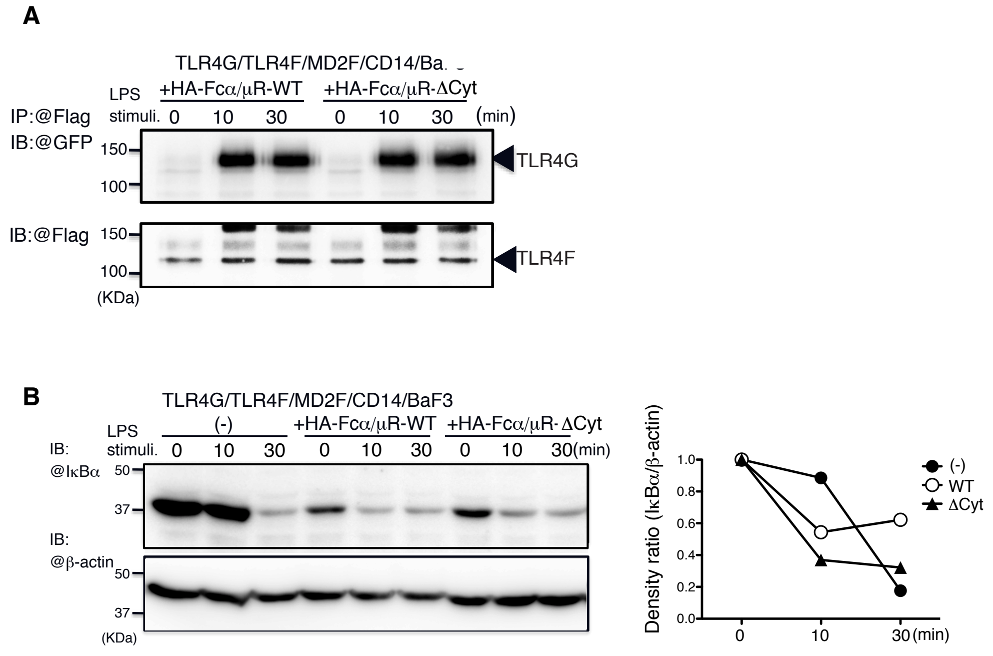

Supplementary Figure 4. Fcα/μR does not require cytoplasmic portion for enhancement of LPS-induced TLR4 oligomerization and NF-κB signaling. Analyses for Ba/F3 cells stably expressing Flag-TLR4, TLR4-GFP, Flag-MD-2, and CD14 together with or without Fcα/μR-WT or Fcα/μR lacking cytoplasmic portion (ΔCyt). (**A**) Immunoprecipitation and immunoblot, as indicated. ▲; TLR4F (Flag-TLR4), TLR4G (TLR4-GFP), as indicated. (**B**) After stimulation of Ba/F3 transfectants with LPS, immunoblotted with indicated Abs. The graph shows the kinetics of densities of each band. Data are representative of three independent experiments.

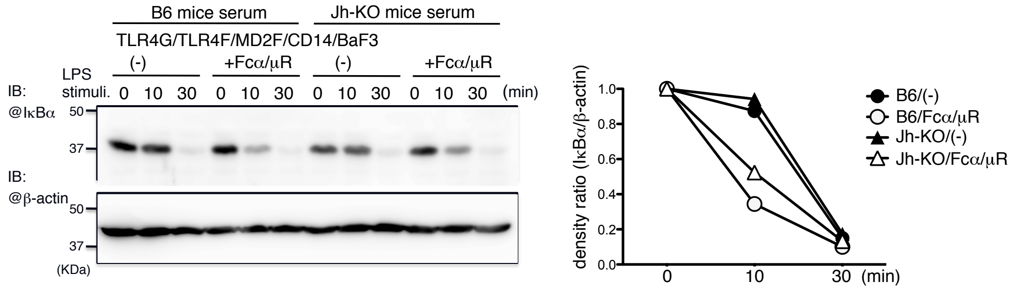

Supplementary Figure 5. Fcα/μR does not require ligand binding for enhancement of LPS-induced NF-κB signaling *in vitro*.

Analyses for Ba/F3 cells stably expressing Flag-TLR4, TLR4-GFP, Flag-MD-2, and CD14 together with or without Fcα/μR. After stimulation with LPS in the presence of mouse serum from Jh-KO or control WT mice, as indicated, immunoblotted with indicated Abs. The graph shows the kinetics of densities of each band. Data are representative of three independent experiments.

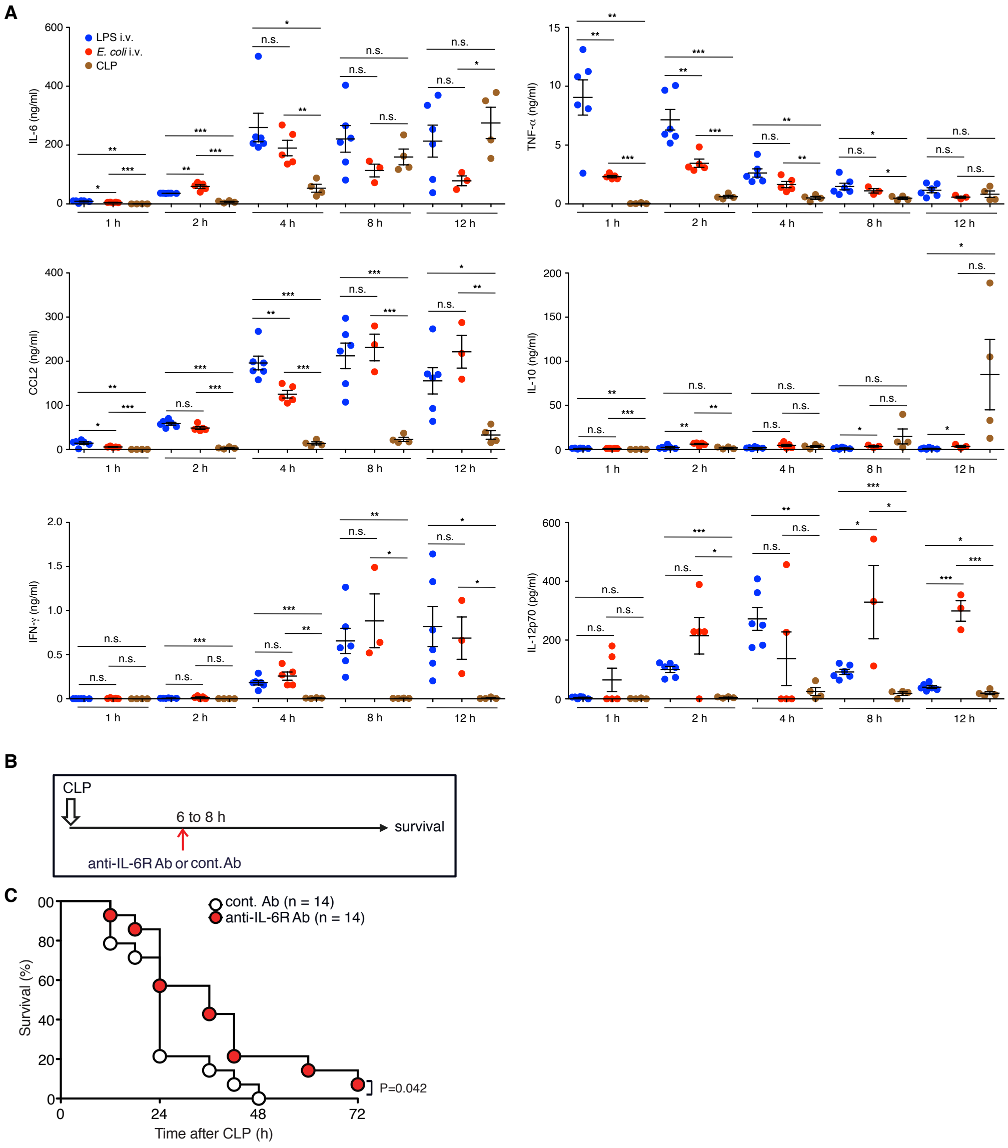

Supplementary Figure 6. Analyses of cytokine and chemokine productions and neutralization of IL-6 in sepsis.

(**A**) Cytometric beads array (CBA) analyses for serum cytokines and chemokines in sepsis induced by injection of LPS or *E. coli* or by cecum ligation and puncture (CLP). (**B, C**) Experimental procedure of antibody injection to CLP. Mice were subjected to CLP, injected with anti-IL-6R Ab or control Ab 6-8 h after CLP, and

monitored for survival rate. Data are pooled of two experiments and the total numbers of mice are indicated. The log-rank test was used for survival.

Supplementary Figure 7. Full-size scans of western blots.

A (Fig. 6B/anti-Flag)

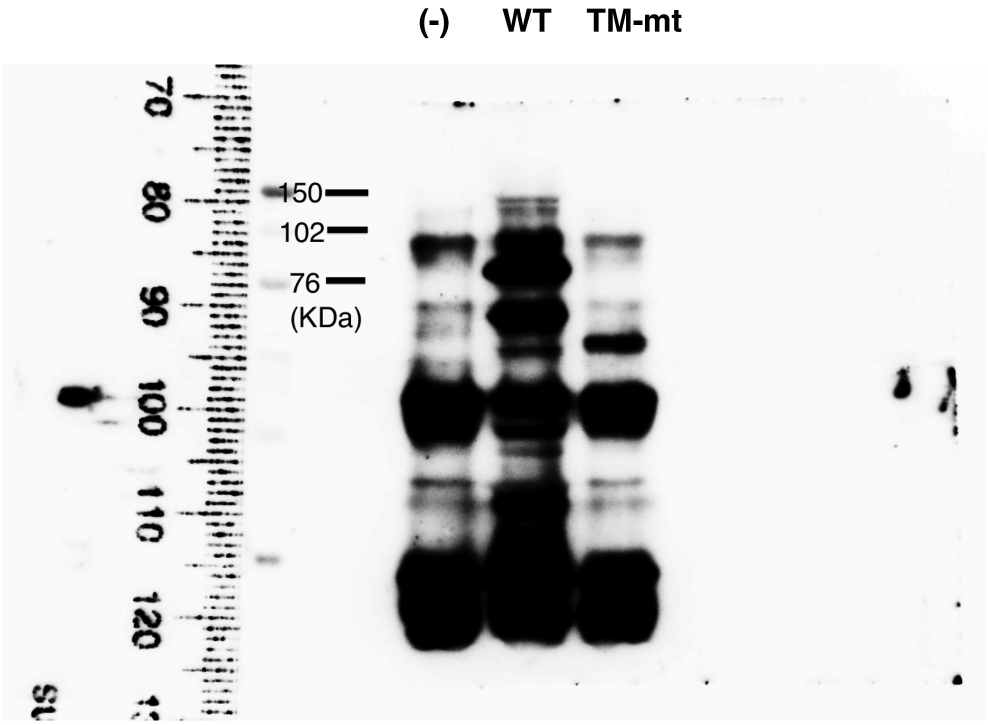

B (Fig. 6B/anti-HA)

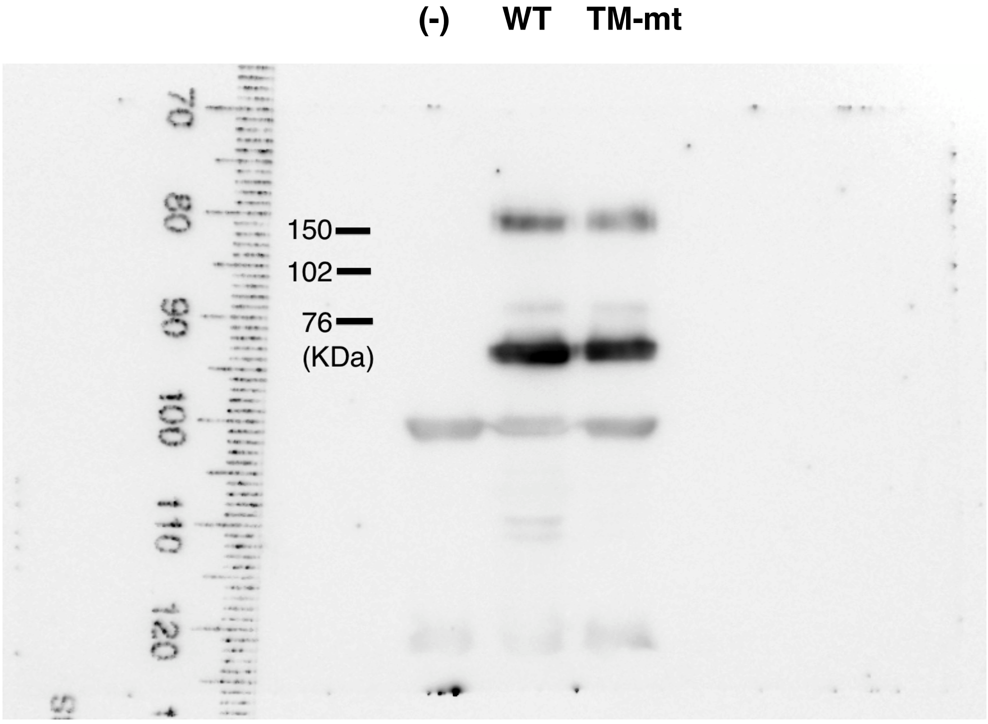

C (Fig. 6C/anti-Flag)

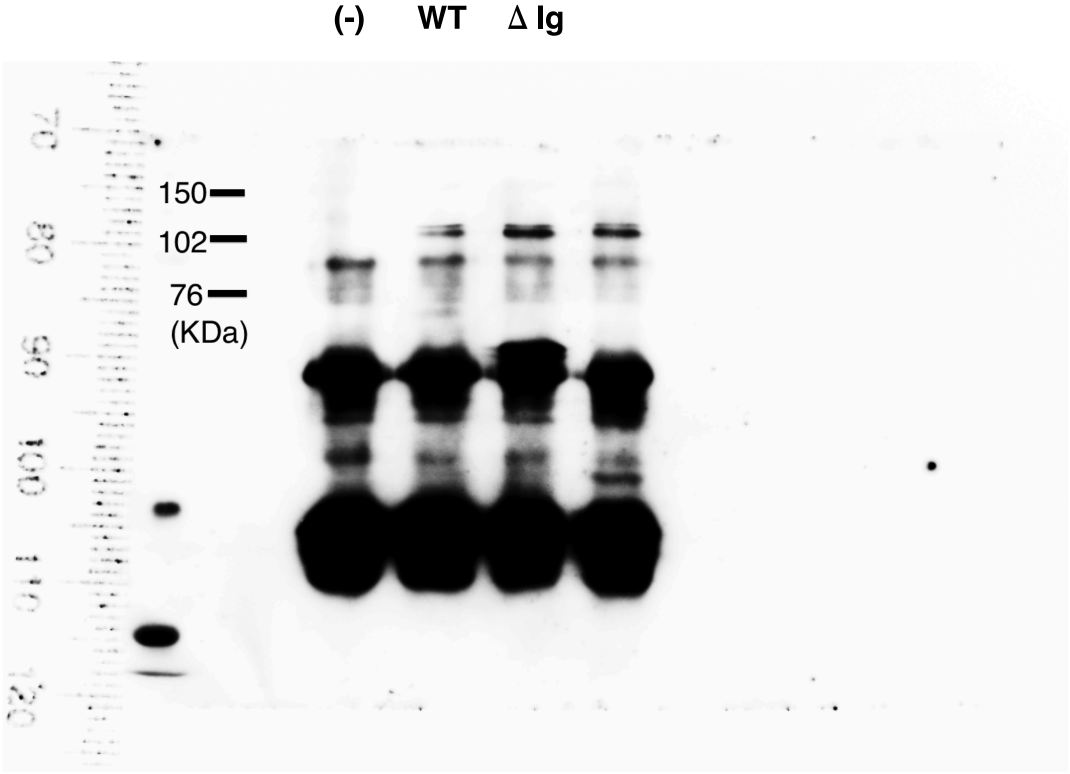

D (Fig. 6C/anti-HA)

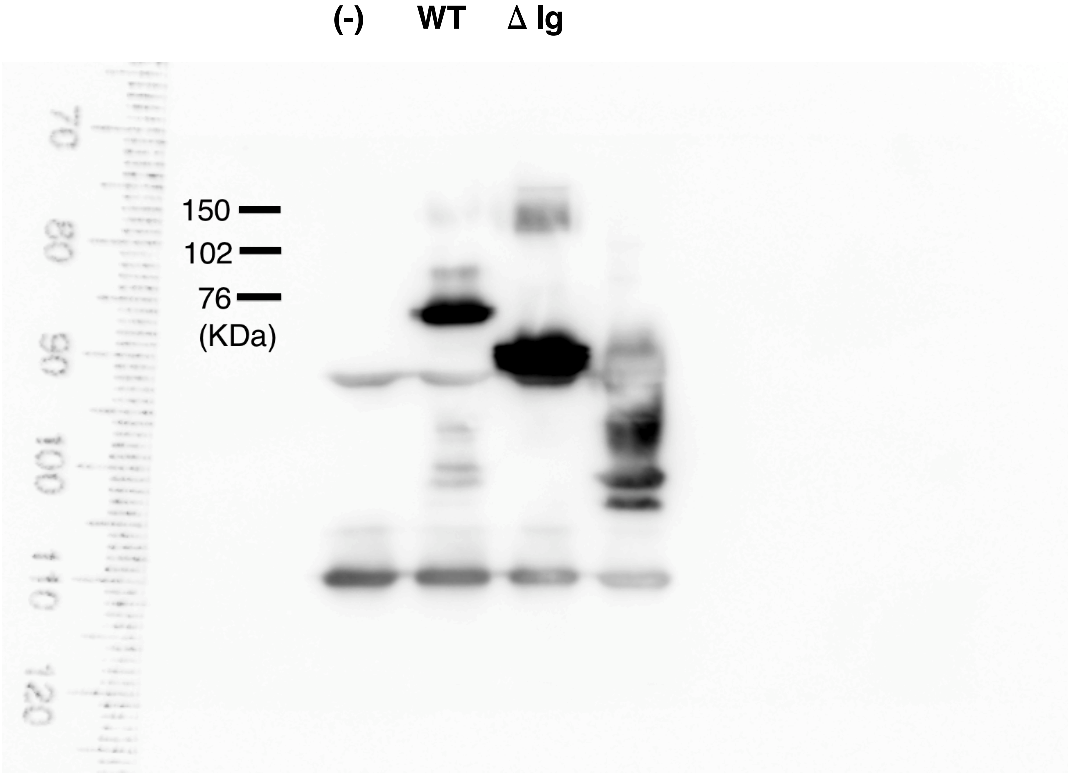

E (Fig. 6C/anti-Flag)

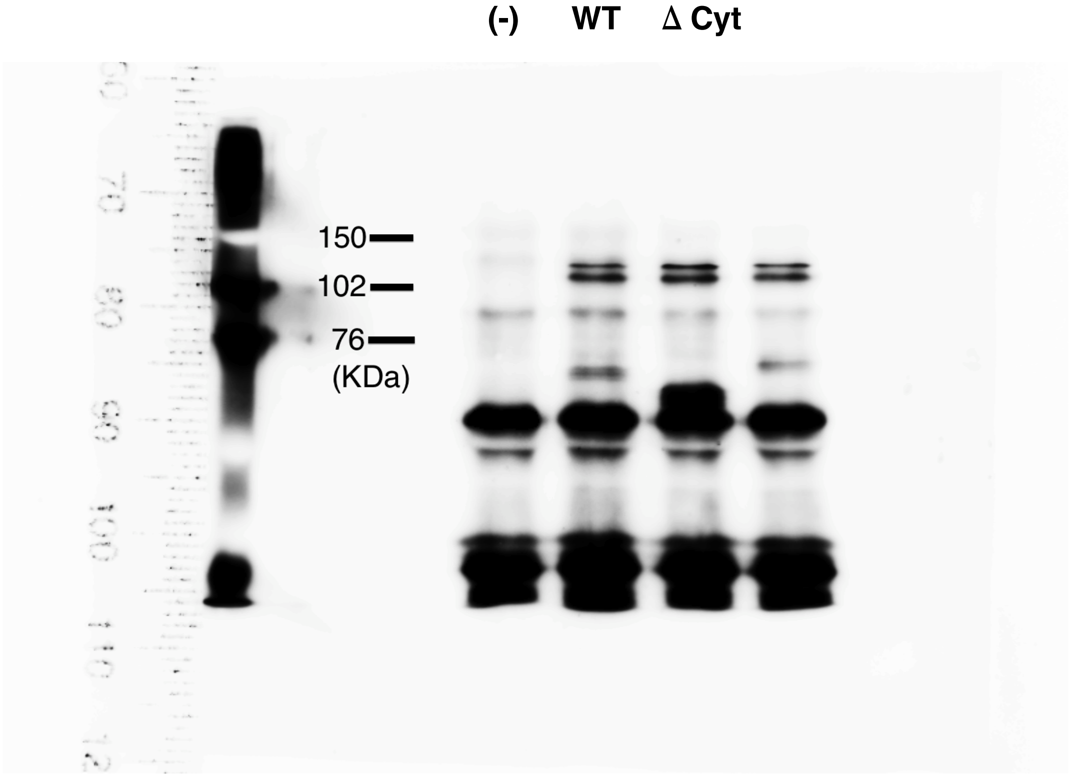

F (Fig. 6C/anti-HA)

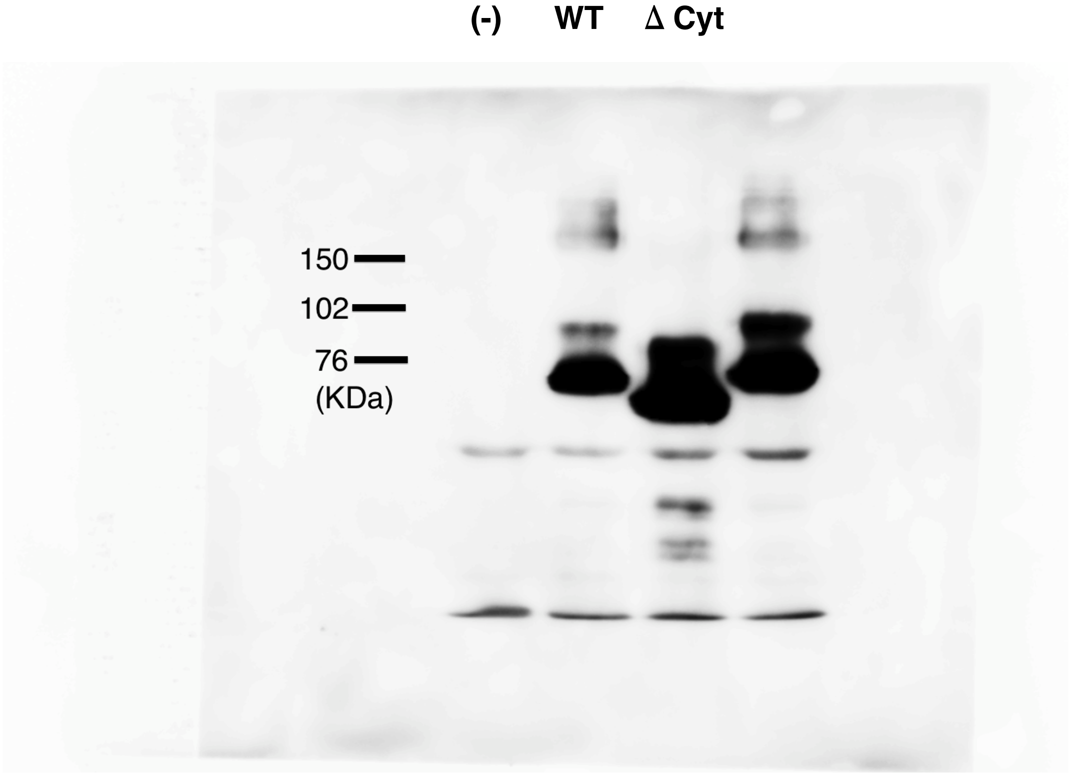

G (Fig. 6D/anti-GFP)

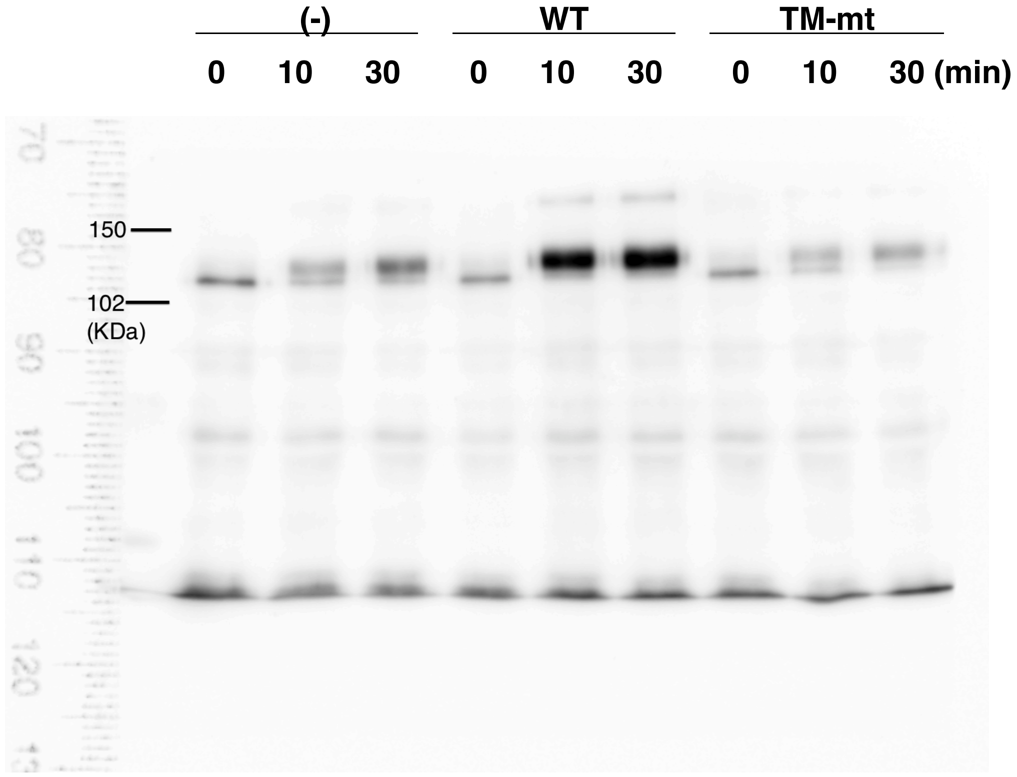

H (Fig. 6D/anti-Flag)

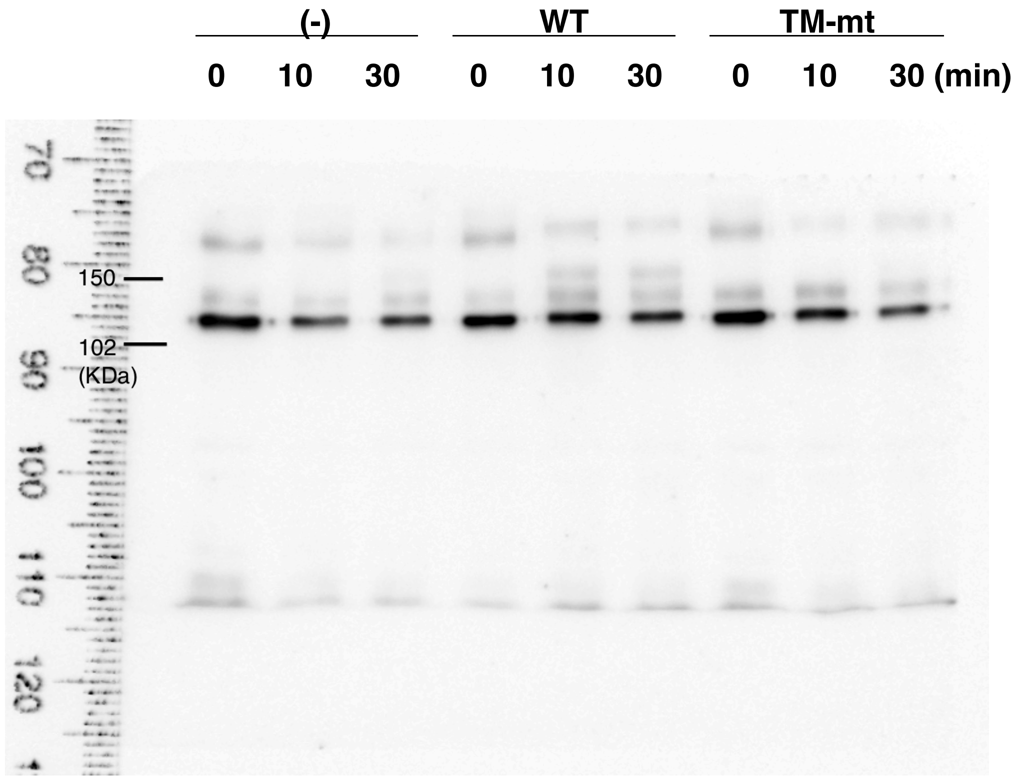

I (Fig. 6F/anti-IκBα)

(-)				WT				TM-mt			
0	10	30	60	0	10	30	60	0	10	30	60 (min)

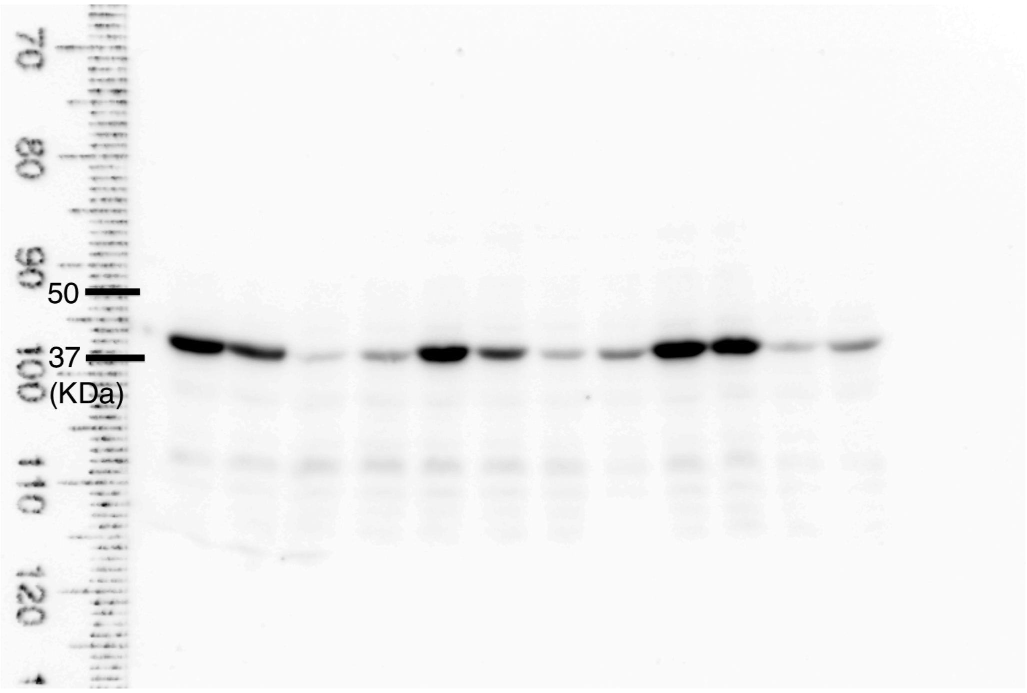

J (Fig. 6F/anti-β-actin)

(-)				WT				TM-mt			
0	10	30	60	0	10	30	60	0	10	30	60 (min)

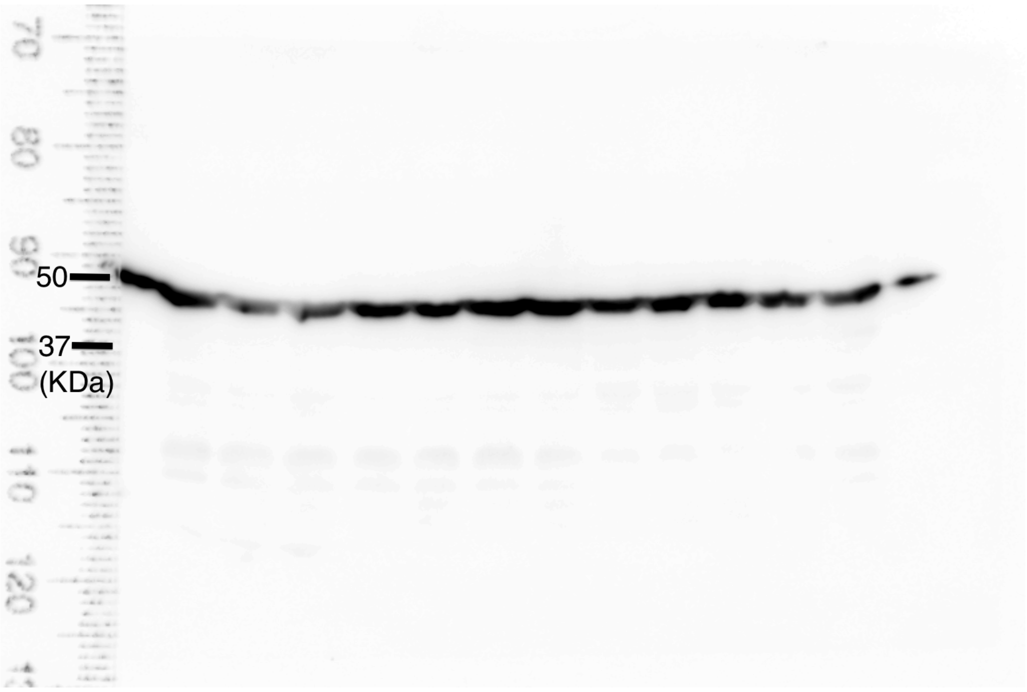

K (Fig. 6G/anti-IκBα)

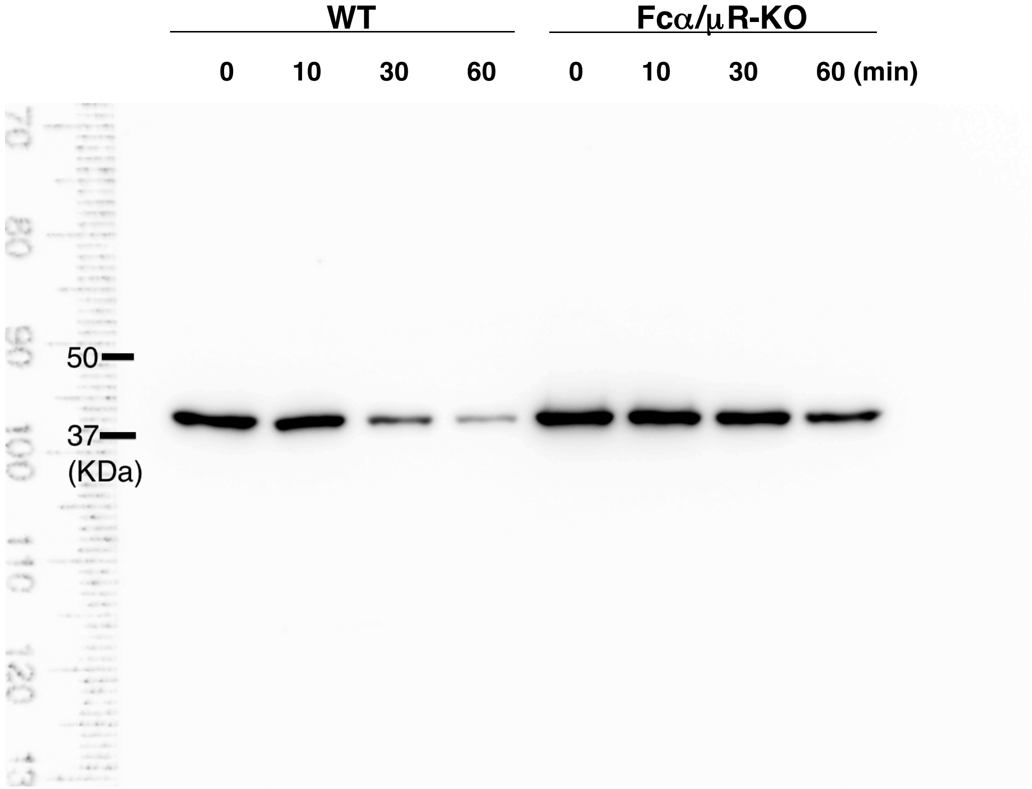

L (Fig. 6G/anti-β-actin)

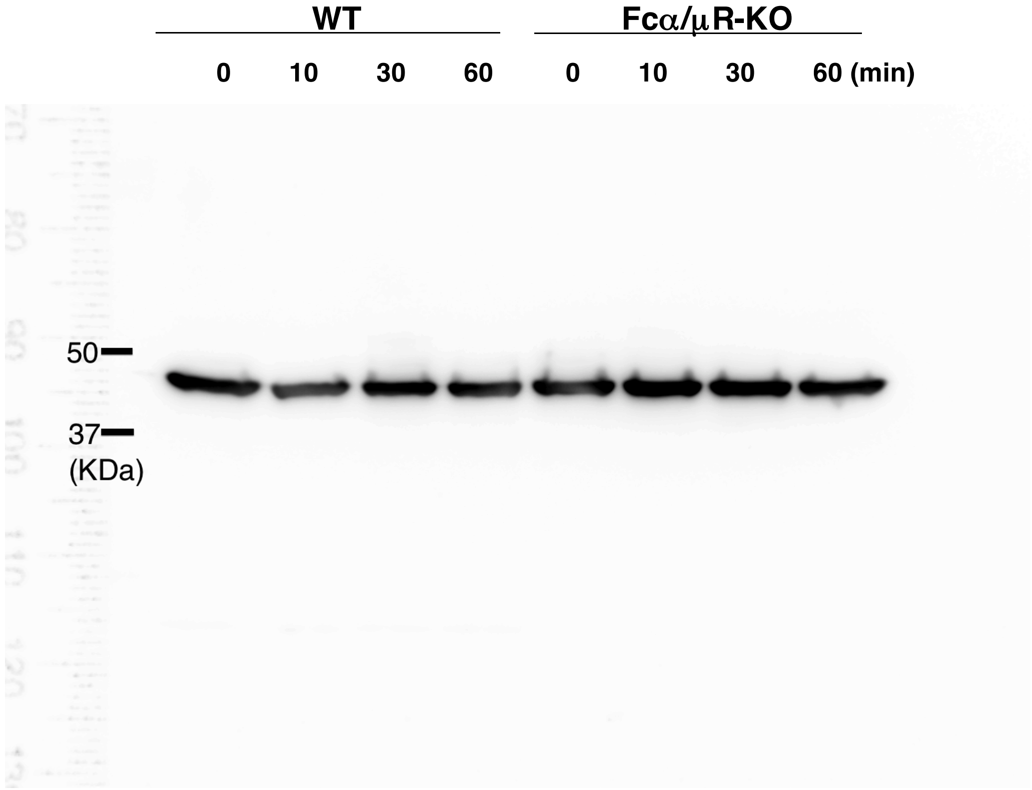

M (S. Fig. 3A/anti-Flag)

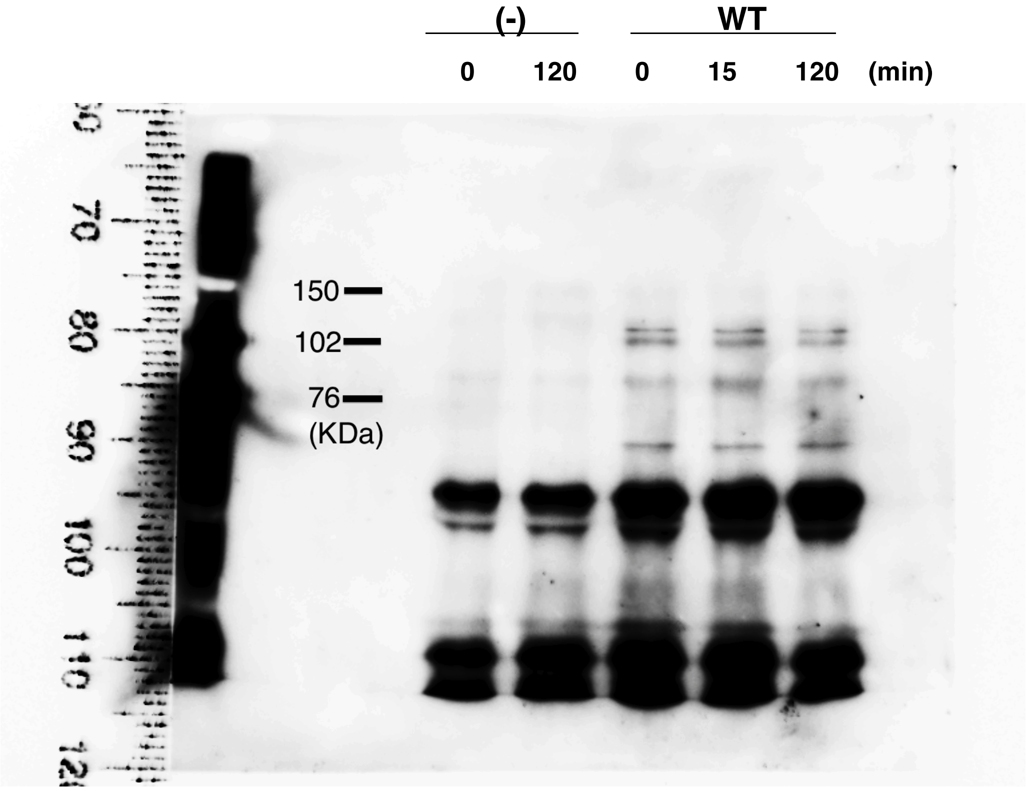

N (S. Fig. 3A/anti-HA)

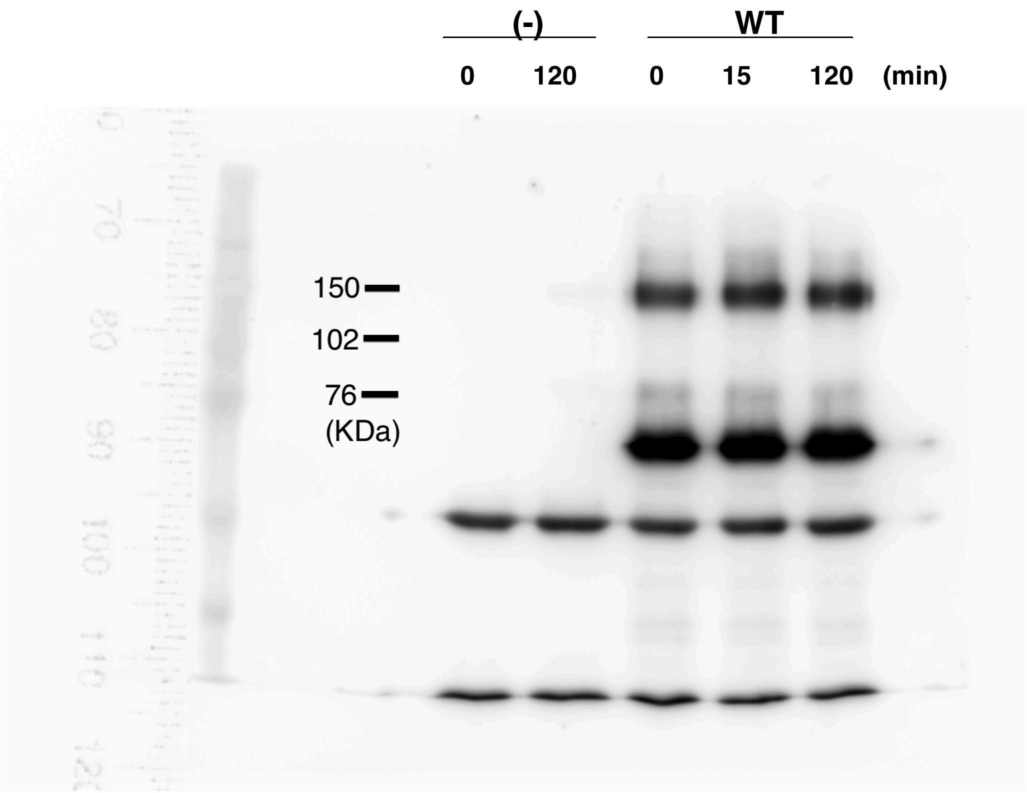

O (S. Fig. 4A/anti-GFP)

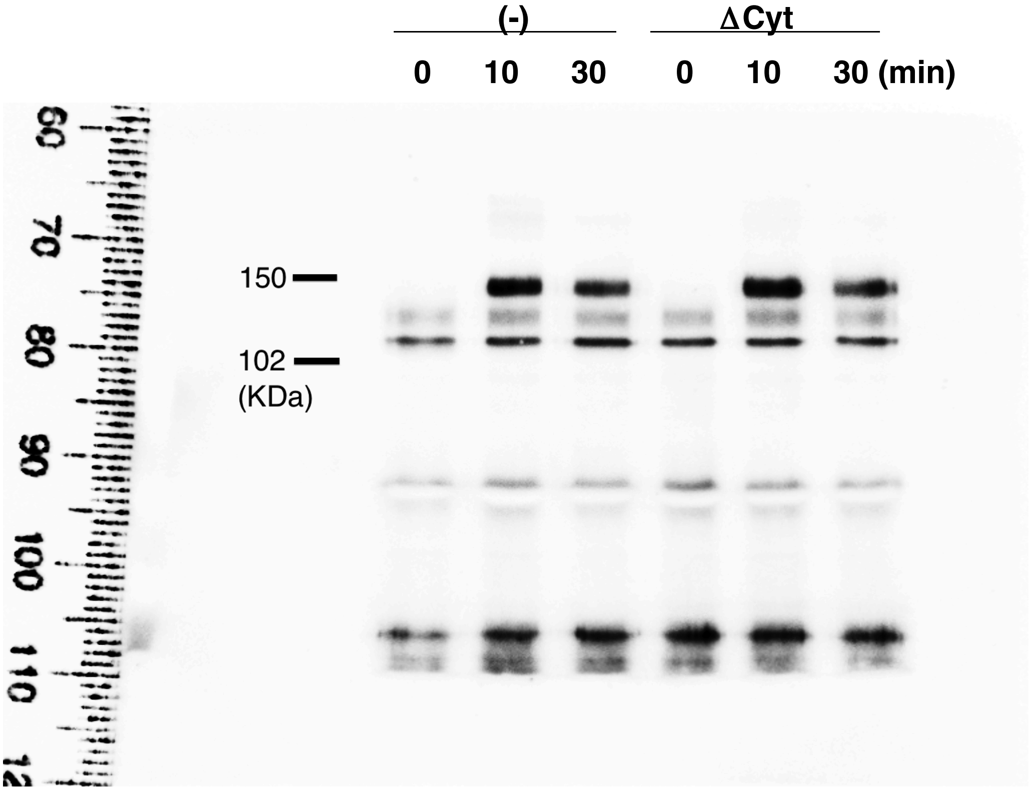

P (S. Fig. 4A/anti-Flag)

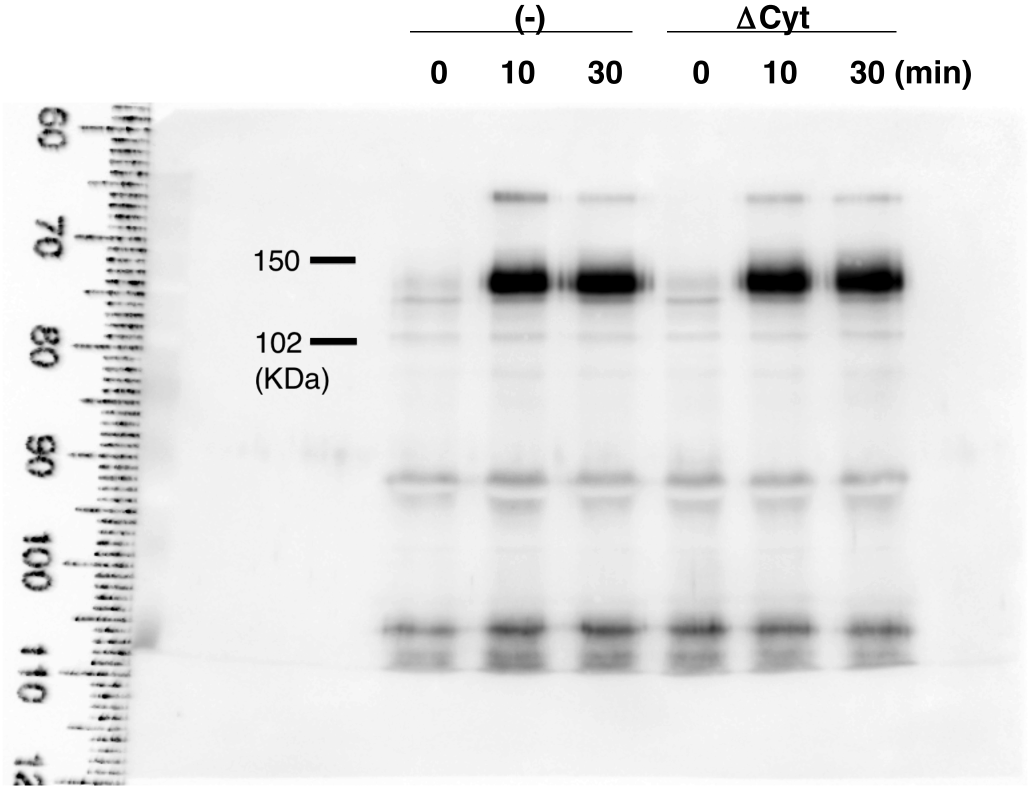

Q (S. Fig. 4B/anti-anti-IκBα)

(-)			WT			ΔCyt		
0	10	30	0	10	30	0	10	30 (min)

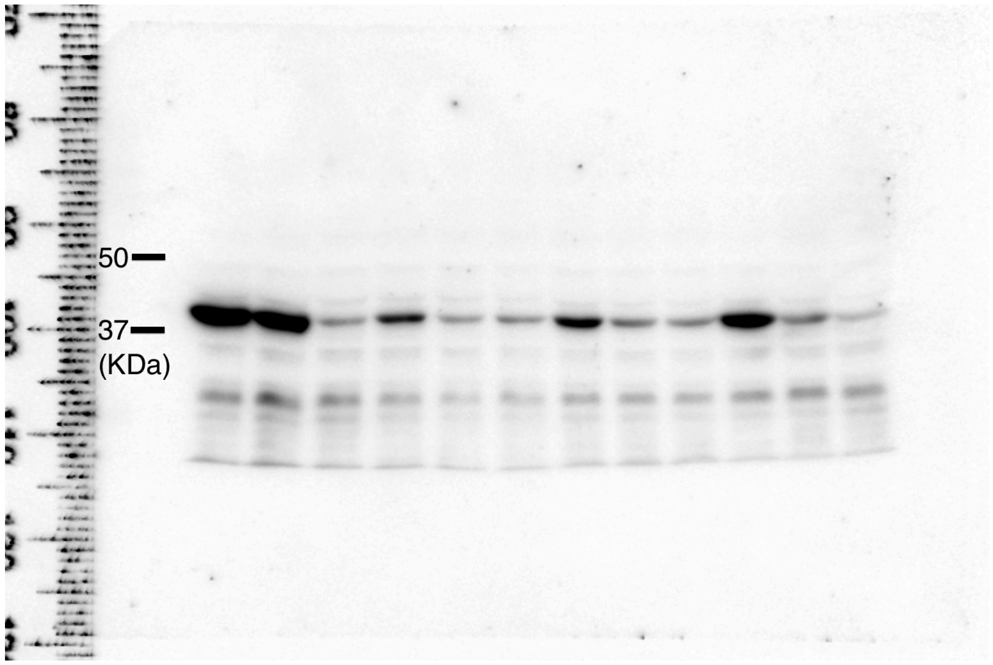

R (S. Fig. 4B/anti-β-actin)

(-)			WT			ΔCyt		
0	10	30	0	10	30	0	10	30 (min)

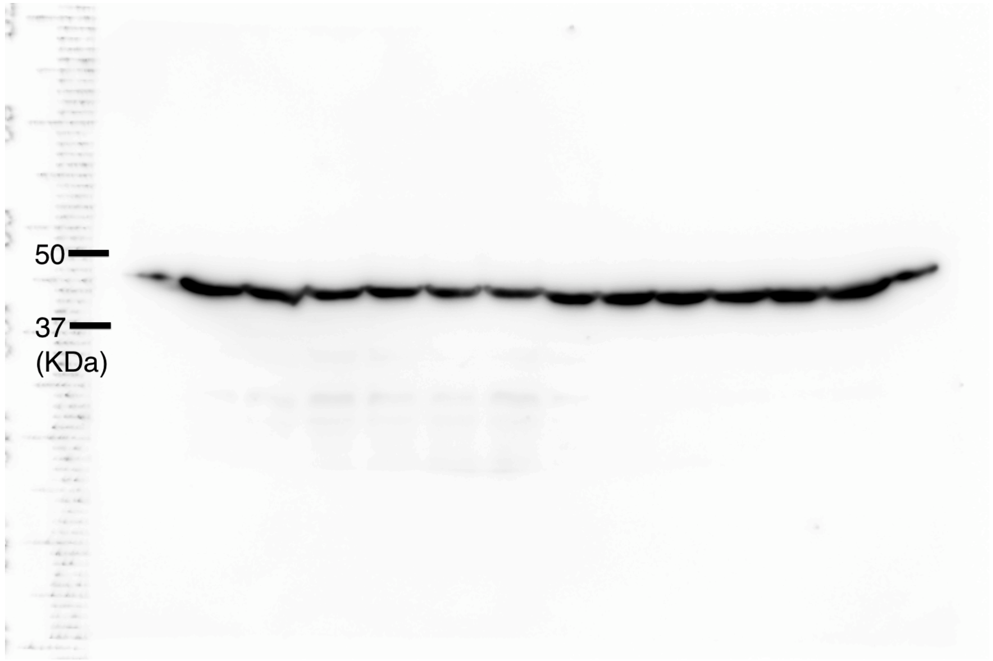

S (S. Fig. 5/anti-anti-IκBα)

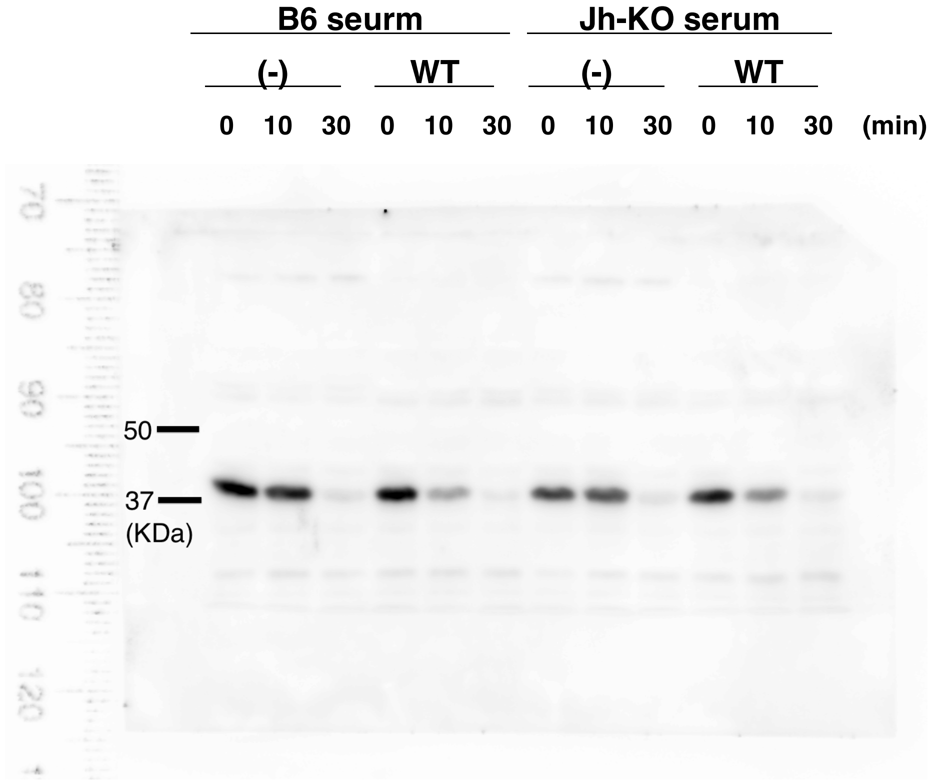

T (S. Fig. 5/anti-β-actin)

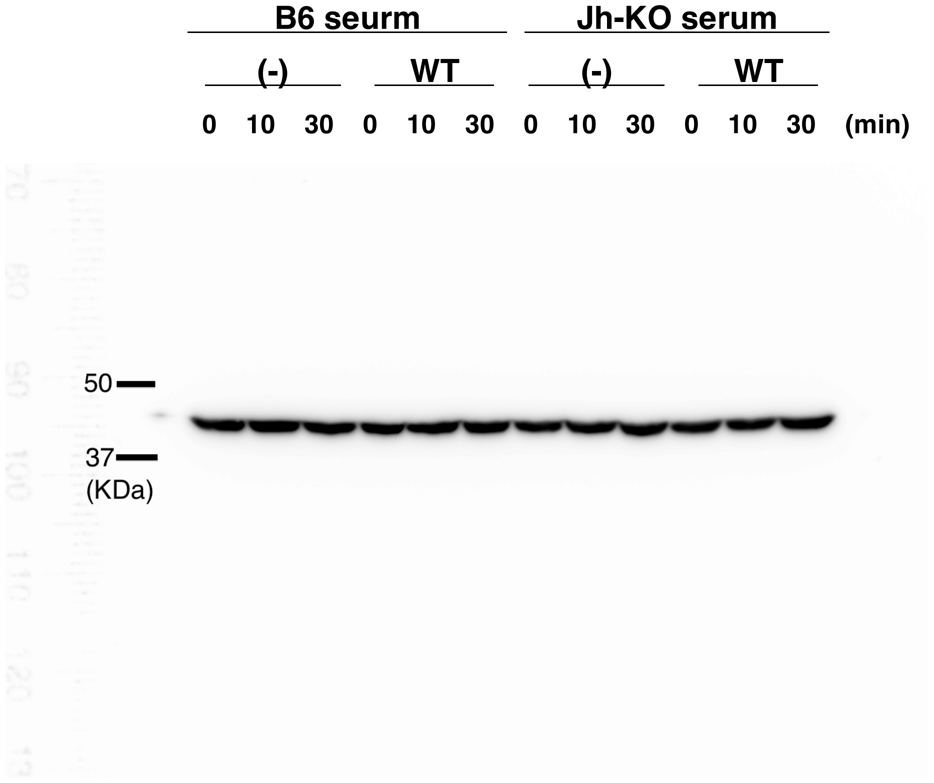

	Percentage					Cell number				
	MZB-WT (n = 4)		ΔMZB (n = 4)			MZB-WT (n = 4)		ΔMZB (n = 4)		
**Spleen**	mean	SD	mean	SD	p	mean	SD	mean	SD	p
marginal zone B cell (MZB)	2.16	0.35	0.35	0.15	0.0001	1.68	0.57	0.28	0.12	0.0001
follicular B cells (FOB)	33.22	0.35	29.89	7.23	0.1477	25.86	1.29	23.48	4.76	0.1327
immature B cells	4.09	0.49	3.65	0.70	0.1152	3.19	0.40	2.89	0.59	0.1668
macrophage	6.33	1.25	6.74	0.30	0.3229	4.92	0.77	5.38	1.63	0.2747
CD4 T cells	23.44	0.57	25.49	1.10	0.1751	21.55	1.97	23.33	1.71	0.3425
CD8 T cells	12.72	1.47	13.61	0.46	0.1160	9.79	1.05	10.68	1.48	0.1278
Natural killer cells (NK)	2.40	0.24	2.16	0.52	0.2494	1.87	0.56	1.72	0.39	0.3010
neutrophil	3.22	0.97	3.44	0.14	0.3574	2.51	0.61	2.75	0.20	0.3132
dendritic cells (DC)	1.28	0.13	1.31	0.08	0.4129	0.99	0.25	1.04	0.09	0.3448
plasmacytoid dendritic cells (PDC)	0.64	0.08	0.65	0.19	0.4499	0.49	0.11	0.51	0.12	0.4098
	(% of total cells)					(x10e6 cells)				
**Peritoneal cells**										
B1a B cells	6.02	0.67	4.74	1.71	0.0654	1.70	0.15	1.44	0.52	0.1313
B2/B1b B cells	16.21	1.12	12.68	4.30	0.0581	4.54	0.36	3.87	1.24	0.1419
macrophage	15.98	3.86	14.60	3.42	0.3939	4.53	0.90	4.45	0.65	0.4779
	(% of total cells)					(x10e5 cells)				
**Bone marrow**										
Pro-/pre-B cells	9.07	2.23	11.19	2.06	0.0940					
immature/mature B cells	2.37	0.93	3.33	0.91	0.0412					
inflammatory mono. (iMo)	5.89	1.49	5.48	0.37	0.2123					
	(% of total cells)									

Supplementary Table 1. Blood cell populations in MZ B-WT and ΔMZ B mice.

Eight weeks after BM cells transfer, immune cells in the spleen, peritoneum and BM of MZ B-WT and ΔMZ B mice were stained with Abs for cell surface markers and analyzed by flow cytometry. The percentages and cell numbers of the indicated cell populations are shown.
